# Restoration of Vision with Ectopic Expression of Human Rod Opsin

**DOI:** 10.1016/j.cub.2015.07.029

**Published:** 2015-08-17

**Authors:** Jasmina Cehajic-Kapetanovic, Cyril Eleftheriou, Annette E. Allen, Nina Milosavljevic, Abigail Pienaar, Robert Bedford, Katherine E. Davis, Paul N. Bishop, Robert J. Lucas

**Affiliations:** 1 Centre for Ophthalmology and Vision Sciences, Institute of Human Development, University of Manchester, Manchester M13 9PT, UK; 2 Manchester Royal Eye Hospital, CMFT, Manchester Academic Health Sciences Centre, Manchester M13 9NT, UK; 3 Faculty of Life Sciences, University of Manchester, Oxford Road, Manchester M13 9PT, UK

**Keywords:** rhodopsin

## Abstract

Many retinal dystrophies result in photoreceptor loss, but the inner retinal neurons can survive, making them potentially amenable to emerging optogenetic therapies. Here, we show that ectopically expressed human rod opsin, driven by either a non-selective or ON-bipolar cell-specific promoter, can function outside native photoreceptors and restore visual function in a mouse model of advanced retinal degeneration. Electrophysiological recordings from retinal explants and the visual thalamus revealed changes in firing (increases and decreases) induced by simple light pulses, luminance increases, and naturalistic movies in treated mice. These responses could be elicited at light intensities within the physiological range and substantially below those required by other optogenetic strategies. Mice with rod opsin expression driven by the ON-bipolar specific promoter displayed behavioral responses to increases in luminance, flicker, coarse spatial patterns, and elements of a natural movie at levels of contrast and illuminance (≈50–100 lux) typical of natural indoor environments. These data reveal that virally mediated ectopic expression of human rod opsin can restore vision under natural viewing conditions and at moderate light intensities. Given the inherent advantages in employing a human protein, the simplicity of this intervention, and the quality of vision restored, we suggest that rod opsin merits consideration as an optogenetic actuator for treating patients with advanced retinal degeneration.

## Highlights

• Ectopic human rod opsin restores visual functions in advanced retinal degeneration
• Rod opsin has greater sensitivity than current optogenetic strategies
• Rod opsin-treated animals respond to spatial stimuli, flicker, and natural scenes
• As a human protein ordinarily found in retinal tissue, barriers to clinic are minimized

## Teaser

Cehajic-Kapetanovic at al. show that ectopically expressed human rod opsin restores vision in a mouse model of advanced retinal degeneration. The quality of the restored vision compares favorably, especially in terms of sensitivity, with alternative approaches, and using a native human protein reduces barriers to future clinical trials.

## Introduction

Inherited retinal degenerations (retinal dystrophies), such as retinitis pigmentosa, affect 1:2,500 people worldwide. Irrespective of etiology, most affect the outer retina and lead to progressive and permanent loss of photoreception. Severe visual impairment is common in advanced stages of the degeneration, and these conditions are currently incurable. However, despite the loss of outer retinal photoreceptors, inner retinal neurons, including bipolar and ganglion cells, can survive and retain their ability to send visual information to the brain [1, 2]. These neurons therefore, represent promising targets for emerging optogenetic therapies that aim to convert them into photoreceptors and recreate the photosensitivity that has been lost during degeneration [3].

Pioneering work has shown that electrophysiological responses to light can be restored to animal models of retinal degeneration by introducing a variety of optogenetic actuators to the surviving inner retina, including the mammalian photopigment melanopsin [4], prokaryotic photoactivated ion channels or pumps [5–10], synthetic light switches [11–14], and a synthetic photopigment (Opto-mGluR6) [15]. These interventions can also support behavioral light responses including, in some cases, maze navigation or optokinetic reflexes reliant upon detection of spatial patterns or fast temporal modulations (flicker). However, in most cases, these actuators function only under very bright light, and, to date, no clinically achievable optogenetic intervention has recreated spatiotemporal discrimination at commonly encountered light levels.

Here, we set out to determine whether it is possible to recreate vision in blind mice using ectopic expression of a natural human protein, rod opsin. Mammalian rod opsins are readily produced under heterologous expression and can couple to native signaling cascades in several cell types in a light-dependent manner [16–19]. We reasoned that if they did this also in neurons of the inner retina, they could restore photosensitivity, and that several features of this approach could be beneficial for clinical application. First, the use of a human protein, and indeed one ordinarily found in the retina, would minimize the potential for immunogenic adverse effects when applied to patients. Second, as a G protein-coupled receptor, rod opsin has access to mechanisms of signal amplification not available to directly light-gated ion channels and thus could have much higher light sensitivity. Finally, rod opsin has the potential to address the need for sensitivity normalization in vision. Detecting objects in our environment relies upon distinguishing local differences in relative luminance across the huge variation in background light intensity. That is only possible because photoreceptors adjust their sensitivity according to the background light intensity. Achieving that goal for optogenetic photoactivators is challenging, but ectopically expressed rod opsin could theoretically do so via two mechanisms. On the one hand, its G protein signaling cascade could show dynamic desensitization. On the other, because rod opsin bleaches upon light exposure, the effective concentration of pigment should be inversely proportional to the background irradiance. The associated reduction in sensitivity is well described for cone photoreceptors where it is termed “bleaching adaptation” [20, 21].

We expressed human rod opsin in surviving inner retinal neurons of a mouse model of aggressive retinal degeneration with near complete loss of rod and cone photoreceptors (*rd*^*1*^) by intravitreal administration of clinically approved adeno-associated virus (AAV) vector, AAV2/2. Widespread light-evoked changes in firing were observed in neurons of the retina and dorsal lateral geniculate nucleus (dLGN) in treated mice. These responses could be elicited using physiologically encountered light levels and under natural light-adapted conditions. Behavioral studies indicated that the treated mice had regained the ability to detect modest changes in brightness, relatively fast flickers, spatial patterns, and naturalistic movie scenes.

## Results

### Gene Delivery to *rd*^*1*^ Retina

**Figure 1:**
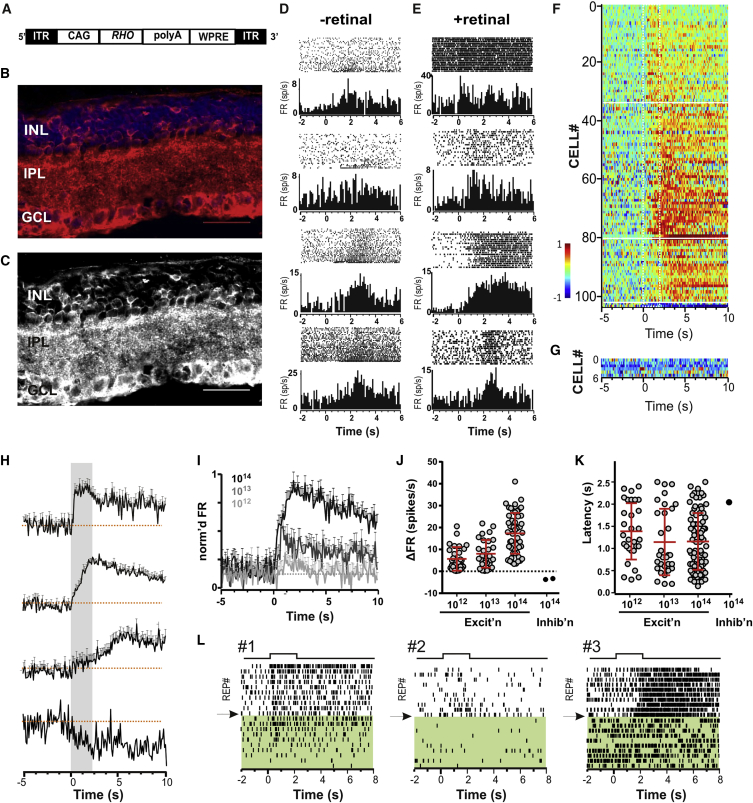
Ectopic Expression of Human Rod Opsin Restores Light Responses in *rd*^*1*^ Mouse Retina (A) Schematic of the DNA expression cassette delivered by AAV2/2 vector to the retina. A human rod opsin coding sequence (*RHO*) is driven by a hybrid CMV enhancer/chickenβ-actin (CAG) promoter. The sequence is flanked by inverted terminal repeats (ITRs) and stabilized by a polyadenylation signal sequence (polyA) and a woodchuck hepatitis posttranscriptional regulatory element (WPRE). (B and C) Exemplar images of a section through an *rd*^*1*^ mouse retina >4 months after intravitreal delivery of vector in (A) in conjunction with glycosidic enzymes. Expression of human rod opsin in cells of the ganglion cell layer (GCL) and inner nuclear layer (INL) and processes in the inner plexiform layer (IPL) are revealed by staining with an α-hRho antibody (red) and counterstaining of nuclei with DAPI (blue) to aid orientation (B). A monochrome version of α-hRho antibody staining in (B) in which rod opsin expression appears in white is shown in (C). Calibration bar = 50 μm. (D and E) Perievent rasters and associated perievent firing rate histograms (PSTHs) for eight representative single units isolated from multi-electrode array (MEA) recordings of *rd*^*1*^-CAG-*RHO* retinas without (D) and with (E) exogenous 9-*cis*-retinal. Each set of rasters depicts spiking activity for 20 sequential presentations of a 2-s white light flash (4 × 10^14^ rod photons/cm^2^/s; interstimulus interval 20 s) starting at time 0. PSTHs below depict mean firing rate in 100-ms epochs across all 20 repeats. In both conditions, units show increases in firing associated with light presentation (from 0 to 2 s), but these are most pronounced for the first few trials (lower traces in raster) in (D), indicating bleaching, while inclusion of *9-cis*-retinal (E) renders them repeatable across many trials. (F and G) Heatmap representations of mean firing rate across at least 20 presentations of 2-s stimulus (ON at time 0) for 104 units from 5 *rd*^*1*^*-*CAG-*RHO* mice (F) and six units from three control *rd*^*1*^*-*CAG-GFP mice (G) meeting an objective criterion of stimulus-associated change in firing. Color code represents normalized firing rate (−1 and 1 being minimum and maximum firing rate for that unit, respectively). Traces are ordered according to response latency. (H) Population mean (±SEM) normalized firing rate profiles for *rd*^*1*^-CAG-*RHO* units grouped according to response latency (horizontal white lines in F delineate extent of clusters). (I) Mean ± SEM normalized firing rate (mean firing rate from −2 s to 6 s was normalized to maximum and minimum, and the normalized pre-stimulus firing rate (−2 to 0 s) was then subtracted) for all light-responsive units exposed to 2-s pulses (starting at 0 s) at 4 × 10^14^, 4 × 10^13^, and 4 × 10^12^ rod photons/cm^2^/s. (J and K) Distribution of response amplitudes (J; mean change in firing rate) and latencies (K; mean time at which mean firing rate first fell outside 2 SDs of baseline firing) for units in (F) responding with increases (excit’n) or decreases (inhib’n) in firing at 4 × 10^14^, 4 × 10^13^, and 4 × 10^12^ rod photons/cm^2^/s. (L) Perievent rasters for three single units showing firing of three units across multiple repeats of a 2-s light pulse (4 × 10^14^ rod photons/cm^2^/s) without (above) and with (below; shaded in green) application of GABA receptor antagonists (TPMP 25 μM and picrotoxin 50 μM).

We injected a viral vector (AAV2/2) containing a human rod opsin coding sequence under control of a CAG promoter (CAG-*RHO*; Figure 1A) into the vitreous of *rd*^*1*^ mice in conjunction with glycosidic enzymes that increase vector transduction [22]. As predicted for this promoter, when retinas were harvested 4–6 months later, immunolabelling revealed rod opsin in both the ganglion cell layer (GCL) and inner nuclear layer (INL) of all treated *rd*^*1*^ mice (Figures 1B and 1C). Expression was found at uneven density across the retina and was generally higher in GCL than INL (Figure S1A). Staining was absent from a control PBS-injected group (Figures S1B and S1C) and the inner retina of wild-type mice (Figures S1D and S1E). Patchy expression was also confirmed in retinal whole mounts for a reporter gene (GFP) delivered via a control AAV2-CAG-GFP vector (Figure S3A).

### Restoring Light-Evoked Activity in Retinal Ganglion Cells

We tested for restored photosensitivity in CAG-*RHO*-transduced retinas by recording spiking activity from the GCL in vitro using a multi-electrode array. 2-s full-field flashes (interstimulus interval 20 s) of broad-spectrum white light increased spiking in numerous units (Figures 1D and 1E). Rod opsin bleaches upon light exposure, and, as might be expected, these responses dissipated over multiple repeats (Figure 1D) unless the culture medium was supplemented with 9-*cis*-retinal, when they became robustly repeatable (Figure 1E). We applied an objective criterion (see Experimental Procedures) to identify light-dependent changes in firing in these retinal explants. This returned 104 out of 671 single units as “light responsive” in CAG-*RHO*-treated retinas (Figure 1F) but only 6 out of 132 units in untreated *rd*^*1*^ mice (Figure 1G). Closer examination of firing patterns in the six light-responsive units in control retinas provides little confidence that they did indeed respond to the stimulus, suggesting that these rather provide an indication of the false-positive rate of our objective test.

Restored ganglion cell light responses varied substantially in response latency (range 0.15 to 2.5 s at ∼4 × 10^14^ rod-effective photons/cm^2^/s) and amplitude (1.21 to 46.51 spikes/s at ∼4 × 10^14^ rod-effective photons/cm^2^/s; Figures 1H–1K). One-third of light-responsive units (n = 34) increased firing within 500 ms of the appearance of light, with a further 46 units responding between 500 ms and 1 s. However, longer delays were also observed (n = 24), including some units being excited after stimulus termination. A very small number of units decreased firing. Responses were obtained not only at maximum intensity (∼4 × 10^14^ rod-effective photons/cm^2^/s) but also when irradiance was reduced by ×10 or ×100 (Figures 1I and 1J), with 31 and 30 units meeting our objective criterion of responsiveness at the two dimmer irradiances. This sensitivity is equivalent to that reported for Opto-mgluR6 [15] but superior to that of microbial photopigments and synthetic light switches, which generally require irradiances in the range 10^14^–10^17^ photons/cm^2^/s [5–14].

One interesting feature of restored light responses is that stimulus-induced increases in firing were much more numerous than decreases (Figure 1F). Rod opsin shows selectivity for Gα_i/o_ class G proteins in heterologous expression [16–19], and one would therefore expect its primary light response to be inhibitory. Nevertheless, this could produce excitatory responses from retinal ganglion cells if it were to reduce the activity of inhibitory amacrine-cell synapses. Previous studies confirm that such sign inversions can occur in the degenerate retina [9, 15]. To test this possibility, we applied GABAa and GABAc receptor antagonists (TPMP 25 μM and picrotoxin 50 μM) to two retinal preparations. We found that excitatory responses were abolished by this treatment (Figure 1L, right-hand records) with the exception of one unit (Figure 1L, left-hand record). These data imply that the excitatory responses we observe originate primarily with light-dependent disinhibition of ganglion cell firing.

### Characterization of Restored Responses In Vivo

**Figure 2:**
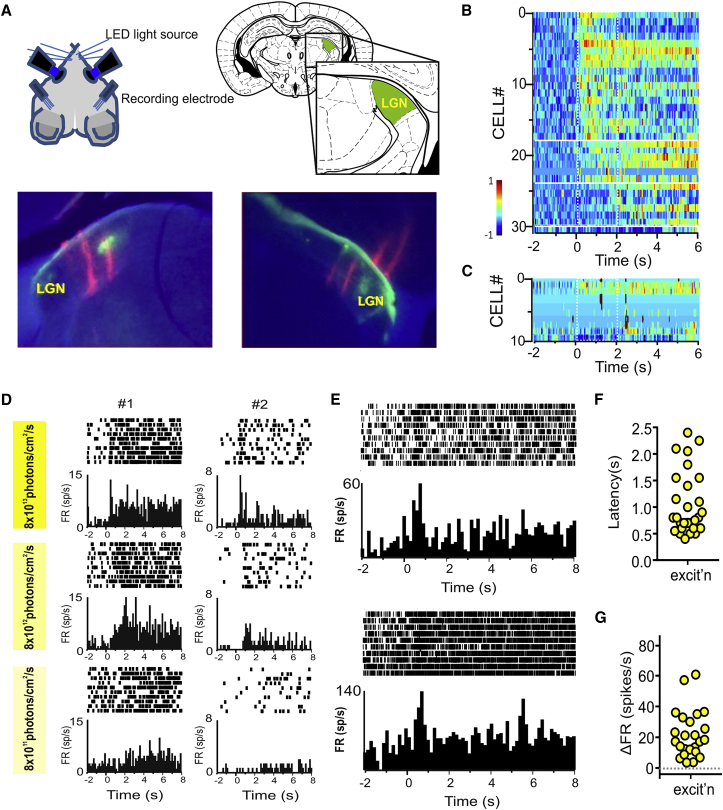
Rod Opsin Expression Driven by the Ubiquitous CAG Promoter Restores Light Responses in Blind *rd*^*1*^ Mouse Thalamus (A) Schematic of recording apparatus allowing presentation of separate light stimuli to each eye and insertion of silicone multi-channel recording electrode probes to the dorsal lateral geniculate nuclei (dLGNs) in either hemisphere. Representative histological sections through the left and right dLGN with DiI tracks (in red) showing path of insertion for recording probes. (B and C) Heatmap representations of mean firing rate across multiple presentations of 2-s stimulus (ON at time 0) to *rd*^*1*^*-*CAG-*RHO* (B) and control *rd*^*1*^*-*CAG-GFP (C) eyes of units showing a significant change in firing associated with stimulus presentation (n = 31 units downstream of 5 treated eyes and n = 10 units downstream of 5 control eyes). Color code represents normalized firing rate (−1 and 1 being minimum and maximum firing rate for that unit, respectively). Traces are ordered according to response latency. (D) Sensitivity response profile (perievent rasters and associated perievent firing rate histograms) for two representative dLGN single units isolated from (B) at three different retinal irradiances: 8 × 10^13^, 8 × 10^12^, and 8 × 10^11^ rod-equivalent photons/cm^2^/s. (E) Light-adapted responses (perievent rasters and associated perievent firing rate histograms) for two representative dLGN units from *rd*^*1*^*-*CAG-*RHO* eyes recorded under light-adapted conditions (retinal irradiance 8 × 10^13^ rod-equivalent photons/cm^2^/s and Michelson contrast 96%). (F and G) Distribution of response latencies (F; time at which mean firing rate first fell outside 2 SDs of baseline for units responding within 2.5 s of lights on) and amplitude (G; mean change in firing rate) for units in (B) responding with increases (excit’n) or decreases (inhib’n) in firing. CAG is a hybrid CMV enhancer/chickenβ-actin promoter. *RHO* is human rod opsin coding sequence.

To determine whether endogenous levels of *cis*-retinal in the degenerate retina were sufficient to allow ectopic rod opsin to function in vivo and how the signal recorded in the retina appeared in the brain, we turned to recording from the dLGN of anaesthetized mice using multi-electrode probes. For these experiments, we used animals in which one eye had been injected with the AAV2-CAG-*RHO* virus and the other with a control GFP virus (AAV2-CAG-GFP; Figure 2A). This enabled us to compare responses to stimuli presented to treated and control retinas in the same individual. We found that 2-s full-field flashes of 410-nm light (estimated retinal irradiance ∼10^14^ rod photons/cm^2^/s) produced many more responses when presented to the treated (Figure 2B) than controls (Figure 2C) eyes. In controls, we found 10 units (out of 736 single units in or around the dLGN) that met our objective criterion of light responsiveness. Several of these had very low baseline firing rate (Figure 2C), making them prone to appear as false positives according to our criterion of responsiveness, while the remainder had very sustained and/or delayed increases in firing as previously described for melanopsin-driven responses [23]. By contrast, stimuli presented to the treated eye induced changes in firing for 31 out of 736 units (Figure 2B). These could be either ipsi- or contra-lateral to the stimulated eye. Bleaching was not a problem for in vivo light responses, which showed robust firing across many repeated trials (Figure 2D) and even to light steps against a background (Figure 2E).

dLGN responses downstream from *rd*^*1*^-CAG-*RHO* retinas were mostly excitatory in nature. Their response duration (0.56 ± 0.84 s; mean ± SD), amplitude, and latency were variable (Figures 2F and 2G), but a cluster of units responded within 500 ms of lights on. There were examples of cells that maintained elevated firing throughout light exposure, and in some cases beyond, while others showed more transient responses (Figure 2D). Responses could be discerned for stimuli at estimated retinal irradiance of 10^14^ and 10^13^, but not 10^12^, photons/cm^2^/s (Figure 2D).

### Restricting Ectopic Expression of Rod Opsin Using a Cell-Specific Promoter

**Figure 3:**
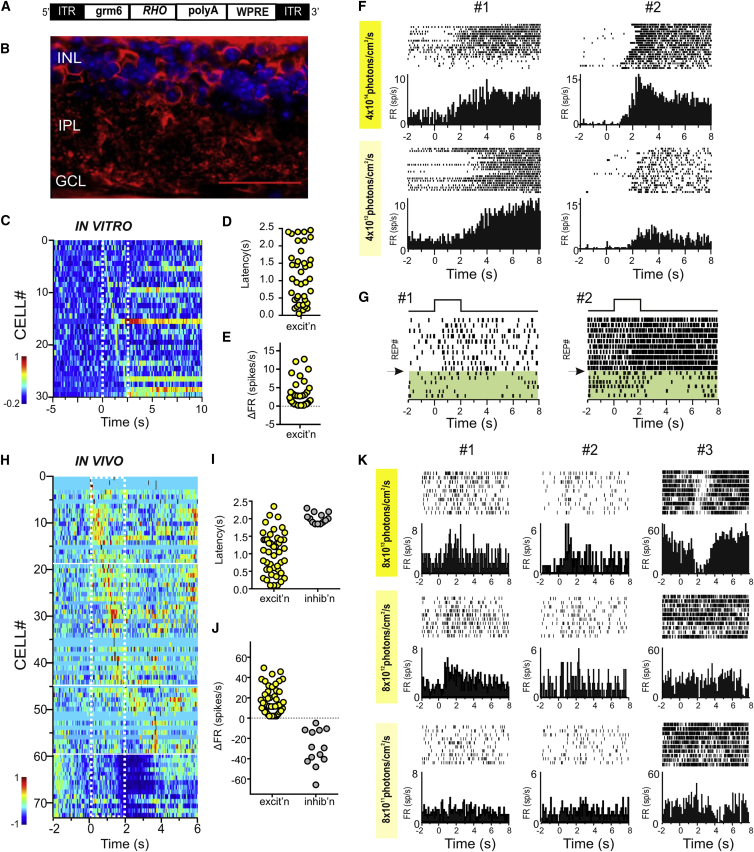
Selective Expression of Rod Opsin Using a Cell-Specific grm6 Promoter Restores Visual Responses in the dLGN of *rd*^*1*^ Mice (A) Schematic of the DNA expression cassette delivered by AAV2/2 vector to the retina, comprising *RHO* under the ON-bipolar cell-specific (grm6) promoter flanked by ITRs and stabilized by polyA and WPRE. (B) Exemplar image of a section through an *rd*^*1*^ mouse retina >4 months after intravitreal delivery of viral vector in (A) in conjunction with glycosidic enzymes. Expression of human rod opsin in cells of the INL and processes in the IPL are revealed by staining (red) with an α-hRho antibody and counterstaining of nuclei with DAPI (blue). Calibration bar = 50μm. (C) Heatmap representations of mean firing rate across multiple presentations of 2-s stimulus (ON at time 0) for 30 single retinal units from two *rd*^*1*^*-*grm6-*RHO* mice showing a significant change in firing associated with stimulus presentation. Color code represents normalized firing rate (−1 and 1 being minimum and maximum firing rate for that unit, respectively). Traces are ordered according to response latency. (D and E) Distribution of response latencies (D; time at which mean firing rate fell outside 2 SDs of baseline for units responding within 2.5 s of lights on) and amplitude (E; mean change in firing rate) for units in (C) responding with increases (excit’n) in firing. (F) Sensitivity response profile (perievent rasters and associated perievent firing rate histograms) for two representative retinal single units isolated from (C) at two different retinal irradiances: 4 × 10^14^and 4 × 10^12^ rod-equivalent photons/cm^2^/s. (G) Perievent rasters for two single units showing inhibition of excitatory responses after application of GABA receptor antagonists (TPMP 25 μM and picrotoxin 50 μM; lower part of raster plots shaded in green). (H) Heatmap representations of mean firing rate across multiple presentations of 2-s stimulus (ON at time 0) for 73 single dLGN units from *rd*^*1*^*-*grm6-*RHO* eyes showing a significant change in firing associated with stimulus presentation. Color code represents normalized firing rate (−1 and 1 being minimum and maximum firing rate for that unit, respectively). Traces are ordered according to response latency. (I and J) Distribution of response latencies (I; time at which mean firing rate fell outside 2 SDs of baseline for units responding within 2.5 s of lights on) and amplitude (J; mean change in firing rate) for units in (C) responding with increases (excit’n) or decreases (inhib’n) in firing. (K) Sensitivity response profile (perievent rasters and associated perievent firing rate histograms) for representative dLGN single units isolated from (H) at three different retinal irradiances: 8 × 10^13^, 8 × 10^12^, and 8 × 10^11^ rod-equivalent photons/cm^2^/s.

A potential problem with untargeted expression of rod opsin is that the pigment will appear in cells that ordinarily would have quite different visual feature selectivity. This could make visual information in the brain incoherent. Therefore, we next selectively targeted rod opsin to ON-bipolar cells (Figure 3A) using an enhancer element derived from the grm6 promoter [24, 25] previously shown to drive expression in this cell type [6, 7, 8, 9, 12]. Viral transduction of a grm6-*RHO* construct resulted in rod opsin expression in cells of the INL across the retina (Figures S2A, S2B, and S2F) often clustered in patches of high transduction (Figure 3B; Figures S2C and S2D). Multi-electrode array recordings of the GCL of two grm6-*RHO*-treated retinas revealed stimulus-associated increases in firing in 30 out of 135 units (Figure 3C). Response latencies (Figure 3D; 1.14 ± 0.778 s; mean ± SD), durations (0.49 ± 0.76 s; mean ± SD), and amplitudes (Figure 3E; 2.8 ± 3.42 spikes/s; mean ± SD) varied significantly. Robust excitatory responses were observed at maximum light intensity (∼10^14^ rod photons/cm^2^/s) and also when the intensity was reduced to ∼10^12^ rod photons/cm^2^/s (Figure 3F). Inhibition of GABAergic signaling abolished these responses (Figure 3G), consistent with the view that they arose primarily from a light-dependent disinhibition of ganglion cell firing.

Electrophysiological responses to light could be readily detected in the dLGN of grm6-*RHO*-treated animals. Thus, when presented with 2-s full-field flashes (410 nm; ∼10^14^ rod equivalent photons/cm^2^/s), numerous units (73 out of 481 units in or around the dLGN) showed a significant change in firing (Figure 3H). Once again, most responses were excitatory, but a number of inhibitory responses (n = 14) were also recorded in this case. Response latencies (Figure 3I; 1.07 ± 0.6; mean ± SD) and amplitudes (Figure 3J; 6.93 ± 9.377; mean ± SD) varied significantly, although many units responded within 500 ms of stimulus onset. Mean (±SD) response duration was 0.41(±0.28) s for increases and 1.21 (±0.75) s for decreases in firing. Responses were apparent at ∼10^14^ and 10^13^ rod photons/cm^2^/s but were less convincing when the stimulus intensity was dropped to 10^12^ rod photons/cm^2^/s (Figure 3K).

### Light-Induced Behavioral Responses

**Figure 4:**
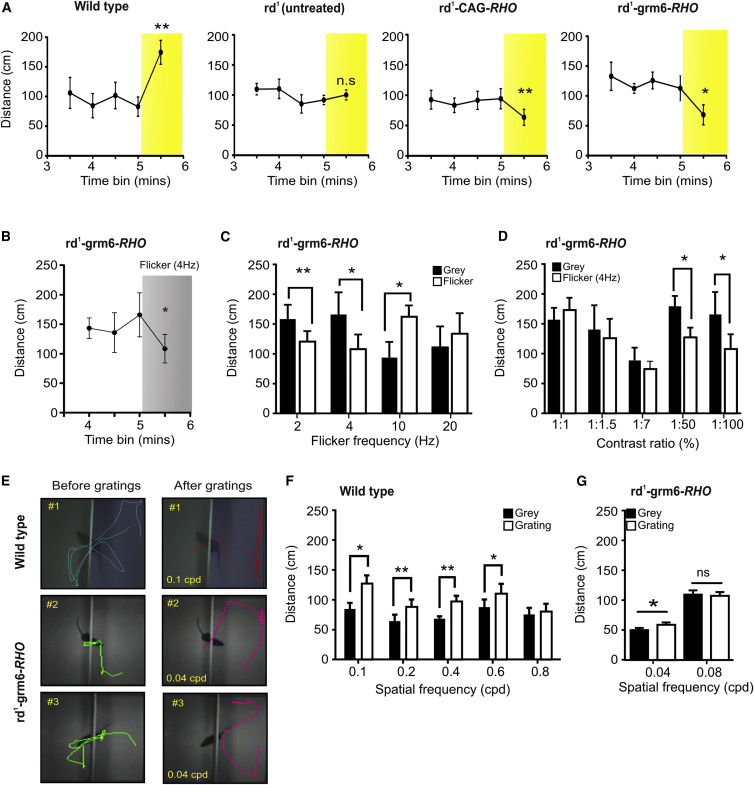
Ectopic Expression of Rod Opsin Restores Visual Behavior in Blind *rd*^*1*^ Mice (A) Open box activity plots for freely moving mice with LCD screens switched from “black” to “white” at time 5 min (illuminance 40 lux; estimated retinal irradiance 1 × 10^12^ rod-equivalent photons/cm^2^/s). (B) Open box activity plot for *rd*^*1*^*-*grm6-*RHO* mice exposed to 4-Hz flicker starting at 5 min (illuminance 20 lux; estimated retinal irradiance 8 × 10^11^ rod-equivalent photons/cm^2^/s. (C and D) Histograms of activity for *rd*^*1*^*-*grm6-*RHO* mice showing distance traveled in 30 s before (black bars) and 30 s after (white bars) presentation of “white” screen at different flicker frequencies (C) and at 4-Hz flicker at different contrast ratios (D). (E) Representative movement trajectories for a wild-type and two different *rd*^*1*^-grm6-*RHO* mice in the open field box in the 30 s before (left) and 30 s after (right) presentation of gratings. (F) Histogram of activity for wild-type mice showing distance traveled in 30 s before (black bars) and 30 s after (white bars) presentation of drifting squarewave gratings (contrast ratio 1:8) at different spatial frequencies. (G) Histogram of change in activity in response to two different spatial frequencies (0.04 and 0.08 cpd) for *rd*^*1*^*-*grm6-*RHO* mice. Sample sizes for data in (A)–(D) are five wild-type, six *rd*^*1*^*-*CAG-GFP, six *rd*^*1*^*-*CAG-*RHO*, and five *rd*^*1*^*-*grm6-*RHO* mice; in (F) eight wild-type; in (G) nine *rd*^*1*^*-*grm6-*RHO*. In all panels, activity is represented by mean ± SEM of the mean distance traveled by each animal in a 30-s time bin; time in min since introduction to testing arena. Two-tailed paired t tests comparing activity before and after stimulus appearance (^∗^p < 0.05, ^∗∗^p < 0.01). For Figures 4B and 4C, two-way RM ANOVA; p < 0.0001 for interaction between flicker frequency and gray versus flicker, post hoc Bonferroni correction p < 0.05 for gray versus flicker at 4 and 10 Hz. For Figure 4F, two-way RM ANOVA; p < 0.01 for gray versus gratings, post hoc Bonferroni correction p < 0.05 at 0.1 and 0.4 cpd.

Next, we asked whether ectopic rod opsin could support visual discrimination. For this purpose, we set out to establish a behavioral test of vision that was higher throughput and less stressful than maze navigation tasks (which in our experience require very long training times for animals with poor vision [26]) and could be used in conjunction with a variety of visual features. Based upon previous light/dark box tests [7, 14, 27, 28] and other reports of behavioral responses to simple visual stimuli [29], we hypothesized that abrupt alterations in the visual scene might induce changes in spontaneous locomotor activity (either increases or decreases) that could be measured objectively with available image analysis software. Mice were placed in a modified light/dark box and allowed free movement between two arenas via an opening in the separating wall. Ordinary LCD computer monitors set to provide corneal irradiance 0.12 W/m^2^ (∼40 lux; retinal irradiance ∼10^11^–10^12^ rod-equivalent photons/cm^2^/s at maximum brightness “white screen” and a contrast ratio of 1:100) were placed behind transparent walls at either end of the arena. We started by asking whether mice could detect a simple luminance step by switching one of the monitors to “white” after the animals had been allowed to explore the box for several minutes with both monitors set to “black.” Wild-type mice responded to the change with an immediate increase in locomotor activity (Figure 4A). This response was absent from control *rd*^*1*^-CAG-GFP mice, while both CAG-*RHO*- and grm6-*RHO*-treated mice responded to the appearance of the white screen with a statistically significant reduction in activity, indicating that they had detected the luminance increment (Figure 4A).

To probe temporal resolution of the restored vision, we investigated whether treated mice could detect the transition from a gray to a flickering screen of equivalent time-averaged irradiance (0.066 W/m^2^). *rd*^*1*^-grm6-*RHO* mice responded to appearance of either 2-Hz or 4-Hz flicker with decreased activity, while 10 Hz drove a significant increase (Figures 4B and 4C; two-way repeated measures [RM] ANOVA; p < 0.0001 for interaction between flicker frequency and gray versus flicker, post hoc Bonferroni correction p < 0.05 for gray versus flicker at 4 and 10 Hz; paired t test p < 0.01 also for 2 Hz). *rd*^*1*^-CAG-*RHO* responded only to the 2-Hz flicker, while *rd*^*1*^-CAG-GFP controls showed no behavioral response in this paradigm (Figure S4A). Using the 4-Hz flicker, we next explored the contrast sensitivity of the flicker detection by reducing the difference in brightness between white and black elements of the flicker (Figure 4D). We found that *rd*^*1*^-grm6*-RHO*-treated mice continued to respond when the contrast ratio was reduced from 1:100 to 1:50, but not 1:7 or lower (Figure 4D).

We used a different cohort of *rd*^*1*^*-*grm6-*RHO* mice to assess spatial acuity for the restored vision. In this case, we asked whether there was a change in locomotor activity associated with the switch from a uniform gray screen to a drifting grating (black:white contrast ratio = 1:7.5; stimuli matched for irradiance). We started by applying this paradigm to wild-type mice to confirm its suitability for our purpose. Appearance of these gratings induced increases in locomotor activity in wild-types at frequencies ≤0.4 or 0.6 cycles per degree (cpd) (Figure 4E, #1; first trial and Figure 4F average of seven trials; two-way RM ANOVA; p < 0.01 for gray versus gratings, post hoc Bonferroni correction p < 0.05 at 0.1 and 0.4 cpd; paired t test p < 0.05 also for 0.2 and 0.6 cpd). Importantly, this finding is consistent with published estimates of spatial acuity in mice from optokinetic and maze navigation methods [30, 31]. We tested treated mice first with a considerably lower grating frequency (0.04 cpd; equivalent to viewing 15-cm bars at 60-cm distance). We found that the grating induced an increase in activity in *rd*^*1*^-grm6*-RHO* mice (Figure 4E, #2 and #3). Across the population of treated mice, this approached statistical significance for the first single trial (p = 0.05) and was statistically significant (p < 0.05) over five (Figure 4G) or ten repeats (p < 0.05, data not shown). *rd*^*1*^-grm6-GFP mice showed no response to this stimulus (data not shown). When tested with a finer grating (0.08 cpd) neither *rd*^*1*^-grm6*-RHO* (Figure 4G) nor *rd*^*1*^-grm6-GFP (data not shown) mice showed a significant change in activity.

### Visual Responses to Naturalistic Scenes

**Figure 5:**
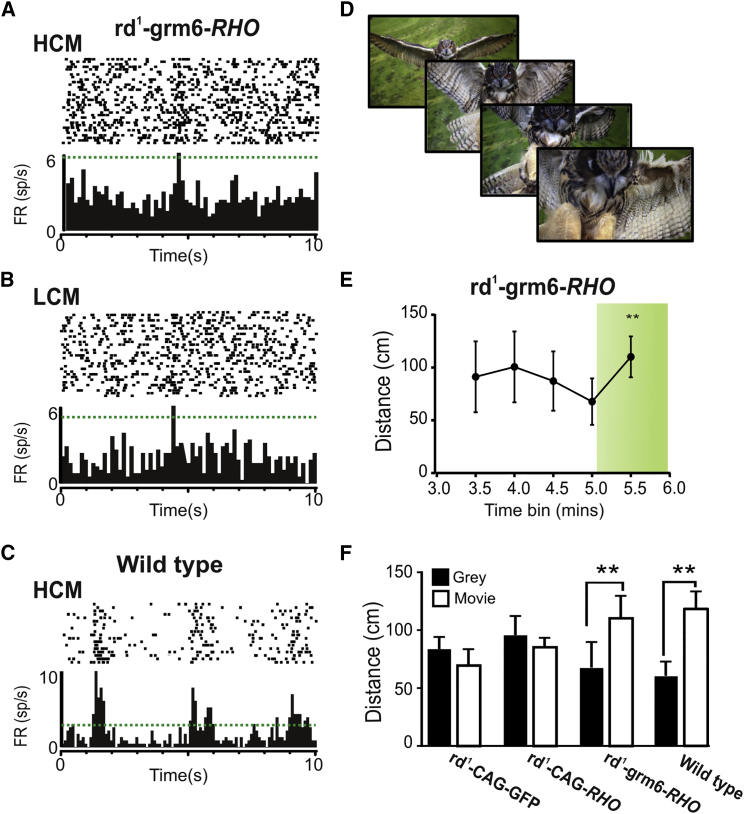
Rod Opsin Restores Visual Behavior in Response to Natural Scenes (A and B) Perievent rasters and associated perievent firing rate histograms for a dLGN unit to multiple presentations of a 30-s naturalistic movie (mice moving in an open arena in horizontal view; mean estimated retinal irradiance 1 × 10^13^ rod-equivalent photons/cm^2^/s) to an *rd*^*1*^*-*grm6-*RHO* eye. (A) and (B) show presentations of the high-contrast movie (HCM; black:white contrast ratio ≈ 1:100) and low-contrast movie (LCM; contrast ratio reduced 1:50), respectively. Horizontal line on histograms shows the 99% confidence interval for firing rate across the movie presentation; note the increase in firing above this line at the same time point for both movie presentations. (C) Firing pattern of a representative dLGN unit from a wild-type mouse exposed to the HCM is presented for comparison. (D) Example frames from a naturalistic movie featuring a swooping owl presented to mice in a behavioral arena. (E) Open box activity plots for *rd*^*1*^*-*grm6-*RHO* mice presented with a naturalistic swooping owl movie starting at 5 min (shaded in green; estimated retinal irradiance 8 × 10^11^ rod-equivalent photons/cm^2^/s). (F) Histogram of activity (mean ± SEM distance traveled by each animal) for *rd*^*1*^*-*CAG-GFP (n = 6), *rd*^*1*^*-*CAG-*RHO* (n = 6), *rd*^*1*^*-*grm6-*RHO* (n = 5), and wild-type (n = 10) mice showing distance traveled in 30 s before (black bars) and after (white bars) presentation of the swooping owl movie. Two-tailed paired t tests comparing activity before and after stimulus appearance (^∗∗^p < 0.01).

The ability of *rd*^*1*^*-*grm6-*RHO* to distinguish spatial patterns at contrast ratios (1:7.5) well within those experienced in natural scenes [32] led us to ask whether ectopic rod opsin might allow discrimination of more naturalistic scenes. We recorded electrophysiological activity in the dLGN across multiple repeats of a 30-s movie comprising mice moving around an open arena [33]. In both *rd*^*1*^*-*grm6-*RHO* and *rd*^*1*^*-*CAG-*RHO* mice, we found units whose firing rate appeared to increase at particular phases on multiple repeats of the movie, suggesting a response to features of the stimulus. However, only one of these from an *rd*^*1*^*-*grm6-*RHO* met an objective criterion of response (Figures 5A–5C). We finally asked whether treated mice could show behavioral responses to a natural movie by presenting a clip of a swooping owl (Figure 5D) to mice in the behavioral test arena. *rd*^*1*^*-*grm6-*RHO* mice responded to this stimulus with a significant increase in activity (Figures 5E and 5F), which was also observed in wild-type mice but was absent in control *rd*^*1*^-CAG-GFP mice or *rd*^*1*^-CAG-*RHO*-treated animals (Figure 5F).

## Discussion

We have found that ectopic expression of human rod opsin is an effective method of restoring vision in blind mice. Using electrophysiological recordings in the retina and visual thalamus, we find that ectopic rod opsin supports reproducible responses to light pulses and steps over a range of intensities typical of our everyday experience. At the single-unit level, restored responses can be excitatory or inhibitory, sustained or transient, mirroring the richness of the visual code seen in wild-type mice. Using a behavioral test, we find that rod opsin-treated mice are able to detect visual stimuli presented using an ordinary LCD visual display unit (VDU) in a dimly lit room. Under these conditions, they can distinguish flicker at a range of frequencies (up to 10 Hz), differences in luminance commonly encountered in visual scenes, coarse spatial patterns, and elements of a natural movie.

The quality of recreated vision reported here for human rod opsin has a number of encouraging characteristics and overall compares favorably with previous approaches. An important feature is its relatively high sensitivity. We find electrophysiological responses at retinal irradiances as low as ∼10^12^ photons/cm^2^/s. This represents a significant improvement in sensitivity compared to previous studies using microbial opsins (thresholds between 10^14^ and 10^17^ photons/cm^2^/s) [5–10], LiGluR/MAG photoswitches (10^15^–10^16^ photons/cm^2^/s) [11, 12], or photoactivated ligands (AAQ at 4 × 10^15^ photons/cm^2^/s [13] and DENAQ at 4 × 10^13^ photons/cm^2^/s) [14] and is similar to the most recent work with the synthetic Opto-mgluR6 receptor (6 × 10^12^ photons/cm^2^/s) [15]. Importantly, this threshold for rod opsin-driven responses falls within the range of irradiances encountered in normal indoor environments.

The relatively high sensitivity of the light responses driven by ectopic rod opsin raises the possibility that this intervention could allow visual discrimination under natural viewing conditions. We employed a new behavioral paradigm to determine the extent to which this was realized. Although developed independently, it is similar to a recently published approach shown to assay cortical vision [28]. At its heart is the prediction that an abrupt change in the visual scene may induce an alteration in behavioral state that can be measured as a change in locomotor activity. As commercially available software can measure mouse locomotor activity in open fields, we hoped that this would provide a simple and objective method to determine whether mice could distinguish between pairs of visual stimuli. That proved to be the case, and in wild-type mice, the new test replicates previous estimates of spatial acuity (Figure 4F) [30, 31]. When applied to treated animals, this behavioral test provides evidence for impressive visual discrimination in *rd*^*1*^*-*grm6-*RHO* mice. These animals showed changes in activity not only to simple luminance increments but also to the appearance of more subtle visual cues including relatively fast flicker (up to 10 Hz) and simple spatial gratings.

Importantly, these responses were elicited under moderate illumination (∼20–150 lux; ∼10^13^ rod equivalent photons/cm^2^/s) and at physiological levels of visual contrast. To our knowledge, this is the first time that a clinically amenable optogenetic intervention has been shown to support spatiotemporal discrimination under such natural viewing conditions. Optokinetic responses to drifting gratings have been recreated using both channelrhodopsin and halorhodopsin, but at much higher irradiances [8, 10]. In a recent study employing opto-mgluR6, such optokinetic responses were recorded at more physiological light levels [15]. However, that work was undertaken in a mouse line in which germline genetic modification was used to express the pigment in all ON-bipolar cells, confounding comparison with the effects of the more clinically relevant viral gene transfer employed here.

The behavioral responses of *rd*^*1*^*-*grm6-*RHO* mice to relatively fast flicker (4 and 10 Hz) indicate that vision in these animals has reasonable temporal resolution and that they can detect stimuli as short as 50 ms. However, it does not follow that they are able to actually resolve the flicker (i.e., detect the train of flashes) at these frequencies. Interactions with head and eye movements could produce apparent modulations at lower frequencies. Moreover, a temporal modulation in irradiance would also be apparent for a photoreceptor integrating over timescales that are not a perfect multiple of the flicker period (although note that the contrast of any such apparent temporal modulation would be strongly negatively correlated with integration period).

One potential advantage of rod opsin therapy is that it relies upon a light-absorbing chromophore (*cis*-retinal) that is naturally produced in the retina. A natural concern, however, is how the availability of the chromophore might be altered in retinal disease. On the one hand, degeneration of photoreceptors (which normally represent a substantial sink for chromophore) might make *cis*-retinal especially abundant in the surviving inner retina. On the other, secondary degeneration of the retinal pigment epithelium (RPE) can be a feature of advanced retinal degeneration, and some forms of dystrophy originate with visual-cycle defects. The effectiveness of rod opsin therapy in *rd*^*1*^ mice (which exhibit RPE dystrophy [34]) argues that in many cases, the degenerate retina would contain sufficient chromophore. In other cases, augmentation with exogenous *cis*-retinal could be considered [35, 36].

In summary, the data presented here indicate that the level of vision recovered by ectopic expression of rod opsin compares favorably with that produced by other optogenetic actuators. Given the simplicity of the intervention and the inherent appeal of a therapy that entails introducing a human protein into a tissue in which it is ordinarily expressed, we suggest that human rod opsin warrants consideration as a method for restoring vision in advanced retinal degeneration.

## Experimental Procedures

See Supplemental Information for details on experimental procedures.

Adult C57BL/6J (wild-type) and C3H/HeJ (*rd*^*1*^) mice were used in this study. All animal experiments and care were conducted in accordance with the UK Animals (Scientific Procedures) Act (1986). Physiological and behavioral experiments were undertaken in mice between 8 and 12 weeks after intravitreal injection of AAV vector administered in isofluorane-anaesthetized mice between 8 and 10 weeks of age. Each eye was injected with 3 μl virus (1 × 10^13^ genomic counts) containing either a rod opsin (AAV2-ITR-CAG-*RHO*-polyA-WPRE-ITR for untargeted expression or AAV2-ITR-grm6-*RHO*-polyA-WPRE-ITR for targeted expression) or GFP (AAV2-ITR-CAG-GFP-polyA-WPRE-ITR for untargeted expression or AAV2-ITR-grm6-GFP-polyA-WPRE-ITR for targeted expression) expression construct, in combination with 0.5 μl of glycosidic enzyme solution containing 0.125 units each of heparinise III and hyaluronan lyase (E.C. 4.2.2.8 and E.C. 4.2.2.1; Sigma-Aldrich). Eyes were retrieved >6 weeks post vector injection, fixed, and cryosectioned before immunohistochemistry and microscopy. For details of gene delivery via AAV vector, histology, immunohistochemistry, and bio-imaging, see Supplemental Experimental Procedures.

### Multi-electrode Array Recordings

Recordings were performed on rod opsin-treated *rd*^*1*^ mice (n = 8) and GFP-injected *rd*^*1*^ controls (n = 3) using a multi-electrode array system (Multi Channel Systems). Light stimuli (2-s full-field flashes of white light, 20-s interstimulus interval, at three different intensities 4 × 10^12^, 4 × 10^13^, and 4 × 10^14^ rod photons/cm^2^/s) were presented by a customized light engine source (Lumencor or Thorlab LEDs). Spike-sorted, single-unit data were further analyzed using Neuroexplorer (Nex Technologies) and MATLAB R2010a (MathWorks).

### In Vivo Electrophysiology

Lateral geniculate nucleus (LGN) recordings were performed on two groups of anaesthetized *rd*^*1*^ mice using a 32-channel probe (Neuronexus). Group 1 (n = 7) had one eye injected with AAV2-CAG-*RHO* and the other with AAV2-CAG-GFP, and group 2 (n = 5) had one eye injected with AAV2-grm6-*RHO* and the other with AAV2-grm6-GFP. Visual stimuli were provided by LEDs (Thorlab λ_max_: 410 nm) and delivered via fiber optic to purpose-made eye cones tightly positioned onto each eye to minimize any potential light leak. Light flashes were delivered according to a light protocol consisting of two parts. Part 1 included flashes from darkness: 2-s light ON, 20-s light OFF, with 10-s offset between each eye. This paradigm was repeated at least ten times at each neutral density (ND) filter. Retinal irradiance ranged from 8 × 10^11^ photons/cm^2^/s at ND2 to 8 × 10^13^ photons/cm^2^/s at ND0. Part 2 of the light protocol involved recording in light-adapted conditions where 5-s steps of light were applied to a steady background illumination at Michelson contrast of 96%. There was a 20-s interstimulus interval and a 10-s offset between two eyes. This paradigm was repeated ten times. Naturalistic movies were presented with a digital mirror device projector (DLP LightCommanderTM, Logic PD), whose intrinsic light engine had been replaced with our own multispectral LED light source containing four independently controlled LEDs (λ_max_ at 405 nm, 455 nm, 525 nm, and 630nm; Phlatlight PT-120 Series (Luminus Devices). For details, see Supplemental Information. We used the same objective criterion to identify light-responsive units in both in vitro and in vivo recordings—that firing rate within 4 s of the start of a 2-s pulse fell >2 SDs outside mean of baseline firing prior to light exposure. Applying this criterion to recordings from control *rd*^*1*^ eyes provides confidence that it returns few false positives; the rate of false negatives is harder to determine. In addition to the responses shown here, it was our impression that in some cases, a light response appeared to have interacted with some underlying oscillatory mechanism, inducing a modest increase in firing around light stimulation and a more substantial change several seconds later. Response duration was estimated by the time over which firing rate fell outside 2 SDs of baseline. A few cells (n = 7 for CAG in vivo; n = 6 for grm6 in vitro; n = 7 for grm6 in vivo) in which the stimulus appeared to have induced a longer-lasting change in baseline firing patterns were not included in this analysis.

### Behavior

Although developed independently, our test is similar to that in a recently published study [28] and shown by them to be a reflection of cortical vision. Using a modification of a light/dark box, mice were allowed free movement between two equal arenas (left and right halves) via an opening in the separating wall. The visual stimuli were displayed from two computer monitors (Acer V173b and either Dell E173FP or ViewSonic matched for power by adjusting screen brightness) facing clear walls of each arena, using a DualHead2Go Digital Edition external multi-display adaptor (Matrox Graphics). A variety of visual stimuli were generated using a custom-written program and displayed on one monitor at a time. For further details on behavioral set up and stimuli used, see Supplemental Information.

## Author Contributions

J.C.-K., A.E.A., C.E., N.M., K.E.D., P.N.B., and R.J.L. designed the research. J.C.-K. performed intraocular injections, retinal histology, LGN recordings, and behavioral experiments. J.C.-K. and C.E. performed MEA recordings. A.P. performed behavioral experiments involving spatial stimuli with assistance from K.E.D., R.B., and N.M. J.C.-K. performed data processing and analysis with assistance from A.E.A. N.M. assisted with histology. J.C.-K., P.N.B., and R.J.L. wrote the manuscript with input from all authors. P.N.B. and R.J.L. supervised the research.

## Supplemental Information

Document S1. Supplemental Experimental Procedures and Figures S1–S4

Document S2. Article plus Supplemental Information
