# Supplementary material for: Restoration of Vision with Ectopic Expression of Human Rod Opsin
Source: Curr Biol. 2015 Aug 17;25(16):2111–22. doi: 10.1016/j.cub.2015.07.029 (PMC4540256; doi:10.1016/j.cub.2015.07.029)
Supplement: Document S1. Supplemental Experimental Procedures and Figures S1–S4 [file mmc1.pdf]

Current Biology

Supplemental Information

Restoration of Vision with Ectopic Expression of Human Rod Opsin

Jasmina Cehajic-Kapetanovic, Cyril Eleftheriou, Annette E. Allen, Nina Milosavljevic, Abigail Pienaar, Robert Bedford, Katherine E. Davis, Paul N. Bishop, and Robert J. Lucas

Figure S1

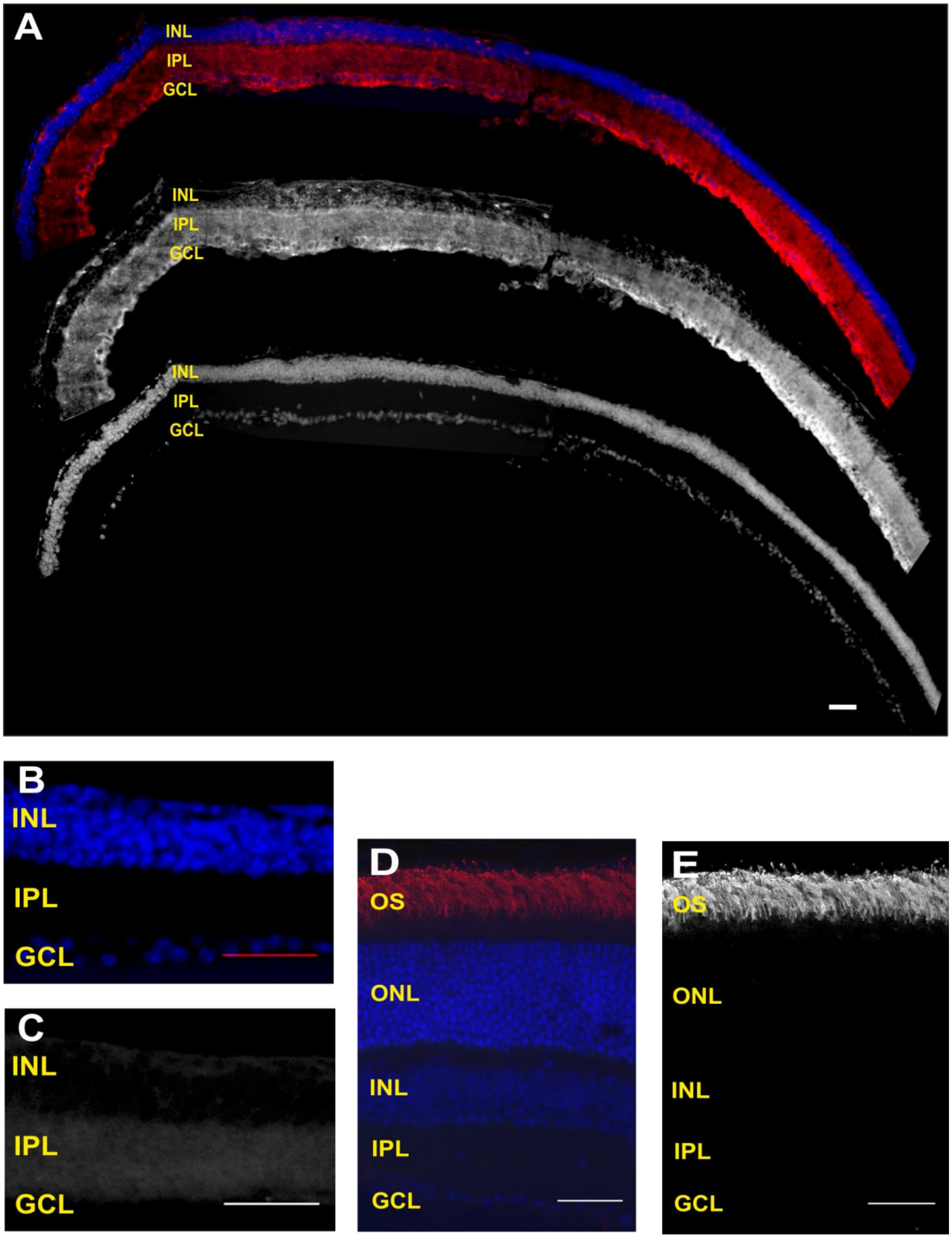
A
INL
IPL
GCL
INL
IPL
GCL
INL
IPL
GCL
B
INL
IPL
GCL
C
INL
IPL
GCL
D
OS
ONL
INL
IPL
GCL
E
OS
ONL
INL
IPL
GCL

Figure S2

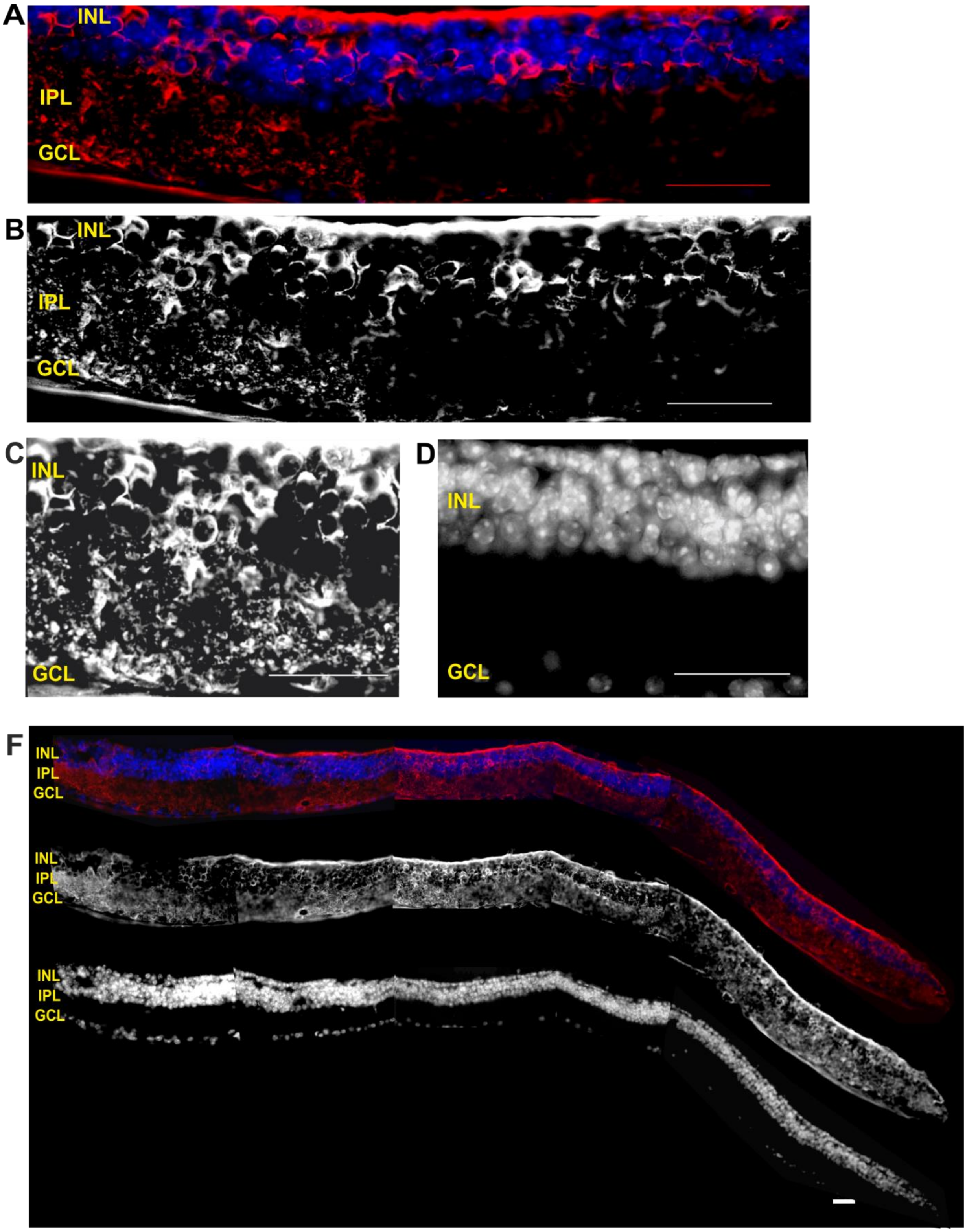
A
INL
IPL
GCL
B
INL
IPL
GCL
C
INL
GCL
D
INL
GCL
F
INL
IPL
GCL
INL
IPL
GCL
INL
IPL
GCL

Figure S3

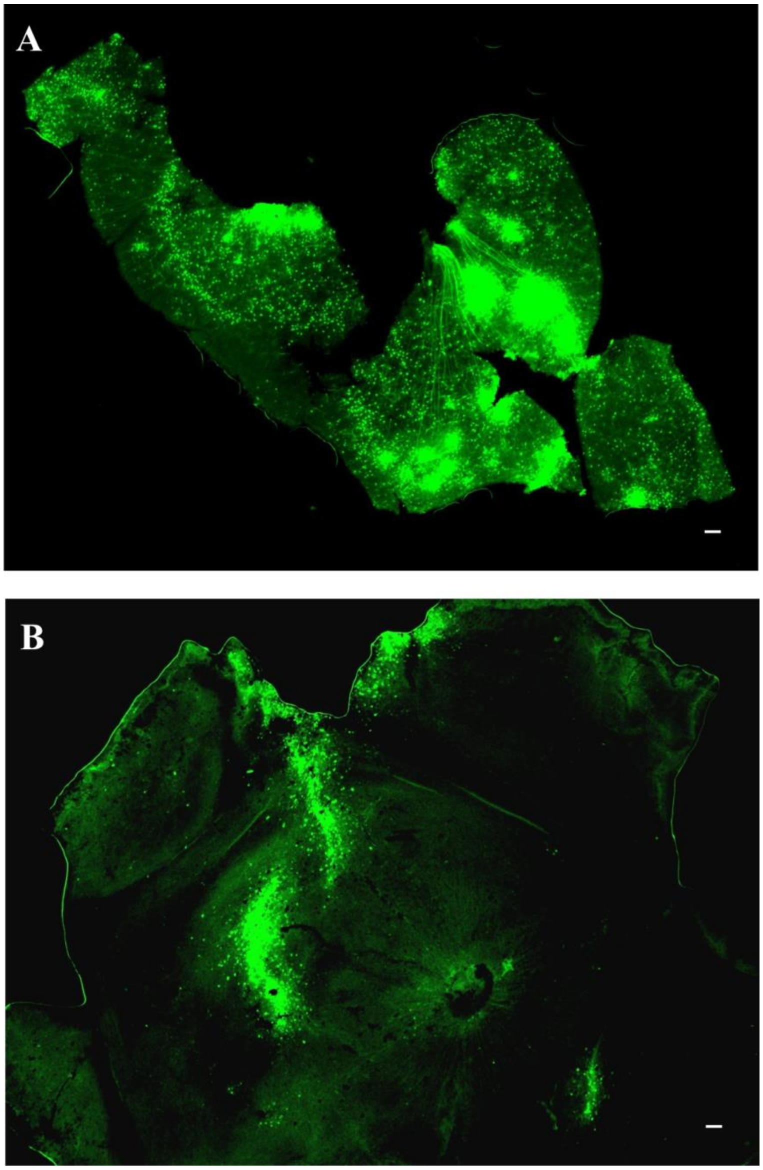

Figure S4

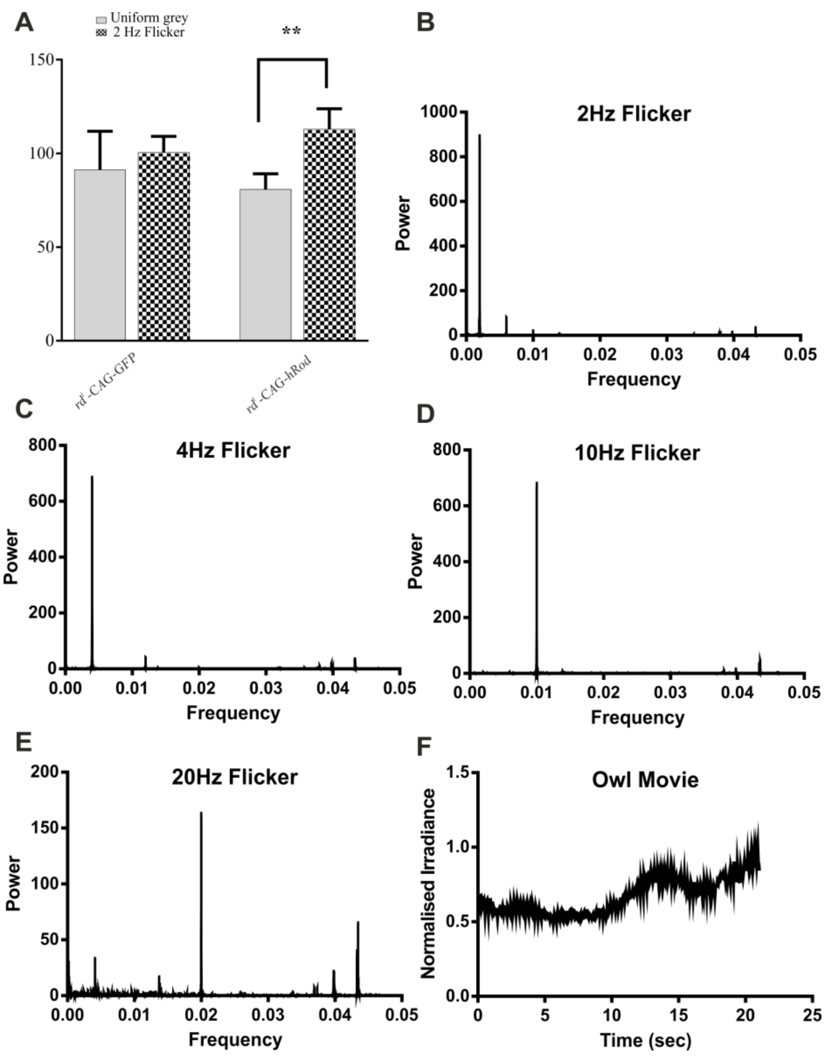
A
Uniform grey
2 Hz Flicker
**
rd-CAG-GFP
rd-CAG-hRod
B
2Hz Flicker
Power
Frequency
C
4Hz Flicker
Power
Frequency
D
10Hz Flicker
Power
Frequency
E
20Hz Flicker
Power
Frequency
F
Owl Movie
Normalised Irradiance
Time (sec)

Figure S1. Rod opsin expression in *rd*[1] retinas using a non-selective (CAG) promoter and control *rd*[1] and *wild-type* stains. Relating to Figure 1.
(A) Exemplar images of a section through an *rd*[1] mouse retina >4 months after intravitreal delivery of AAV2/2-CAG-*RHO* in conjunction with glycosidic enzymes. Uneven density of rod opsin expression is shown after staining with α-hRho antibody (upper image in red; middle image in white, a monochrome version of antibody staining). Nuclei are stained with DAPI (upper image in blue; lower monochrome image in white). Note that this impression of staining across a retinal section was produced by splicing together several high magnification images of smaller portions of the retina. (B to E) images of sections through PBS injected *rd*[1] (B and C) and *wild-type* (D and E) mouse retina showing no staining (B and C) and photoreceptor outer segment (OS) staining (D and E) after treatment with α-hRho antibody; nuclei are stained with DAPI (blue); C and E are monochrome versions of α-hRho antibody staining in (B) and (D) respectively; nuclei are stained with DAPI (blue). INL – inner nuclear layer, IPL- inner plexiform layer, GCL – ganglion cell layer, ONL – outer nuclear layer. CAG - a hybrid CMV enhancer/chickenβ-actin promoter. *RHO* - human rod opsin coding sequence. Calibration bar = 50µm.

Figure S2. Rod opsin expression in *rd*[1] retina using an ON-bipolar specific (grm6) promoter. Relating to Figure 3.
(A) A longer section of the retina presented in Figure 3B >4 months after intravitreal delivery of AAV2/2-grm6-*RHO* in conjunction with glycosidic enzymes showing the extent of rod opsin expression in cells of the inner nuclear layer (INL) and processes in the inner plexiform layer (IPL) after staining (red) with an α-hRho antibody and counterstaining of nuclei with DAPI (blue). (B) A monochrome version of α-hRho antibody staining in (A) clearly depicting rod opsin expression in white. (C) A monochrome version of α-hRho antibody staining in Figure 3B showing a cluster of moderately high rod opsin expression (white). (D) A monochrome version of DAPI staining in Figure 3B. (F) Exemplar images of a section through an *rd*[1] mouse retina >4 months after intravitreal delivery of AAV2/2-grm6-hRho in conjunction with glycosidic enzymes. Uneven density of rod opsin expression is shown after staining with α-hRho antibody (upper image in red) and (middle image in white, a monochrome version of antibody staining). Nuclei are stained with DAPI (upper image in blue and lower monochrome image in white). Note that this impression of staining across a retinal section was produced by splicing together several high magnification images of smaller portions of the retina. GCL - ganglion cell layer. grm6 - ON bipolar cell specific promoter. *RHO* - human rod opsin coding sequence. Calibration bar = 50µm.

Figure S3. GFP localisation in whole mount *rd*[1] retinas following intravitreal injection of AAV2 driving transgene expression under a non-selective (CAG) promoter or an ON-bipolar specific (grm6) promoter. Relating to Figure 1 and Figure 3. Retinal wholemounts in both A (using a non-selective (CAG) promoter) and B (using an ON-bipolar specific (grm6) promoter) depict uneven expression of a marker (GFP) driven by these promoters. Transduced cells are shown in green. Calibration bar = 50µm.

Figure S4. Behavioural activity in treated and control *rd*[1] mice in response to 2Hz flicker and visual stimuli used for behavioural experiments. Relating to Figure 4 and Figure 5. (A) Histogram of activity for *rd*[1]-CAG-GFP (n = 6) and *rd*[1]-CAG-*RHO* (n = 6) mice showing

distance travelled in an open field box 30s before (grey bars) and 30s after (chequered bars) presentation of a 2Hz full field flicker. Activity is represented by mean±SEM of the mean distance travelled by each animal in a 30-sec time bin; time in minutes since introduction to testing arena. Two tailed paired t-tests comparing activity before and after stimulus appearance (** $p < 0.01$). CAG - a hybrid CMV enhancer/chickenβ-actin promoter. *RHO* - human rod opsin coding sequence. (B-F) Flicker and naturalistic movie stimuli were generated using standard LCD monitors. To assess the characteristics of these stimuli we measured irradiance with a photodiode (Advanced Photonix SLD-70 BG2A) connected to a 10-bit analog-to-digital converter of a microcontroller board (Arduino UNO). The sampling rate of acquisition was 100 Hz. The power spectrum graphs show frequency in kHz and power in arbitrary unit. B-E show power spectrum analyses for irradiance traces at 2, 4, 10 and 20Hz. Note the contamination of lower frequency components at 20Hz (as we approach the limit of hardware performance) but not other frequencies. F shows the change in irradiance (normalised to max=1) over the course of a single repeat of the swooping owl movie.

Supplemental Experimental Procedures

Gene delivery via AAV

AAV vector was administered via intravitreal injection in isofluorane anaesthetised mice aged 8 to 10 weeks. Prior to injections, pupils were dilated with tropicamide and phenylephrine. A custom made ultra-fine needle (Hamilton RN needle 34 gauge, supplied by ESSLAB) was attached to a 5µl Hamilton glass syringe and was passed at 45 degrees through the pars plana into the vitreous cavity, carefully avoiding the lens and blood vessels. The injection was performed under a direct visualisation of the needle tip through cover-slipped eyes under an operating microscope (Microscopes Inc., USA). The vectors, rAAV serotype 2 (rAAV2/2, or simply AAV2) expressing rod opsin or GFP under the control of a strong ubiquitous pan-neuronal promoter (CAG) or ON-bipolar cell specific (grm6) promoter were obtained from Vector Biolabs, Philadelphia, USA. The CAG promoter is a fusion of CMV early enhancer and chicken β-actin promoter. The grm6 promoter is a fusion of 200-base pair enhancer sequence of the mouse grm6 gene encoding for ON-bipolar cell specific metabotropic glutamate receptor, mGluR6, and an SV40 eukaryotic promoter. The gene of interest in each case was flanked by inverted terminal repeat (ITR) domains and stabilised by polyadenylation signal sequence (polyA) and a woodchuck hepatitis posttranscriptional regulatory element (WPRE).

Each eye was injected with 3µl virus ($1x10^{13}$ genomic counts) containing either a rod opsin (AAV2-ITR-CAG-*RHO*-polyA-WPRE-ITR for untargeted expression or AAV2-ITR-grm6-*RHO*-polyA-WPRE-ITR for targeted expression) or GFP (AAV2-ITR-CAG-GFP-polyA-WPRE-ITR for untargeted expression or AAV2-ITR-grm6-GFP-polyA-WPRE-ITR for targeted expression) expression construct, in combination with 0.5µl of glycosidic enzyme solution containing 0.125 units each of heparinise III and hyaluronan lyase (E.C. 4.2.2.8 & E.C. 4.2.2.1; Sigma-Aldrich, Dorset, UK). The enzyme solutions were made fresh on the day of injection by dissolving the enzymes in sterile phosphate-buffered saline (PBS). The vector and enzymes were mixed in a syringe immediately before an eye injection and were given in a single combined injection.

Histology

Retrieved eyecups (>6 weeks post vector injection) were fixed in 4% paraformaldehyde (PFA) for 24 hours at 4° C. The tissue was then washed in PBS and further fixed in 30% sucrose in PBS overnight at 4° C. Fixed eyes were cryo-protected in optimal-cutting temperature medium (Raymond A Lamb Ltd., Eastbourne, UK) and frozen at -80° C until further processing. Cryo-protected retinal section were sectioned on a cryostat (Leica, Microsystems) horizontally through the eyecup at 8-10μm thickness from ventral to dorsal side, so that each section contained a complete nasal to temporal cross-section of the retina. Ten-twelve sections were collected on each slide containing sections representative of the entire retina. Slides were stored at -80° C.

For immunohistochemistry, slides were removed from the freezer and allowed to air-dry at room temperature for 1 hour. Sections were permeabilised by immersing slides in PBS with 0.2% Triton for 20 minutes at room temperature. Following this sections were background blocked with PBS with 0.2% Triton X-100 containing 10% donkey serum (D9663; Sigma, UK) for 1 hour at room temperature. Primary antibody (Rabbit Anti-Human Rhodopsin, Abcam, Ab112576) was applied at 1:200 dilution in blocking buffer (PBS with 0.2% Triton X-100 and 2.5% donkey serum) for 3 hours at room temperature. After washing in tween 0.05% PBS, four times for 10 minutes, sections were incubated with secondary antibody (Alexa Fluor® 546 Donkey Anti-Rabbit IgG (H+L) Antibody, Life technologies, lot: 1504518) diluted 1:200 in PBS with 0.2% Triton X-100 and 2.5% donkey serum for 2 hours at room temperature. Slides were then washed four times for 10 minutes in 0.05% tween PBS followed by one final wash with dH_20. After removing excess fluid, slides were mounted with fluorescent mounting media containing DAPI (Vectashield, Vector Laboratories Ltd., Peterborough, UK) to stain cell nuclei. For bio-imaging, sections were analysed under an Olympus BX51 upright microscope using x4, x10 and x20 Plan Fln objectives and captured using a Coolsnap ES camera (Photometrics, Tucson, AZ) through MetaVue Software (Molecular Devices Ltd. Wokingham, UK). Images were taken under specific band pass filter sets and colour-combined images were used for further processing using ImageJ.

Multi-electrode array recordings

Enucleated eyes were placed in a petri dish filled with carboxygenated (95% CO2/5%CO2) aCSF (artificial cerebro-spinal fluid, concentration in mM: 118 NaCl, 25 NaHCO3, 1 NaH2PO4, 3 KCl, 1 MgCl2, 2 CaCl2, 10 C6H12O6, 0.5 L-Glutamine). Retinas were then carefully isolated in diffuse red light under a dissecting microscope and mounted, ganglion cell side down, onto a 60- or 256-channel multi-electrode array (Multi Channel Systems, Reutlingen, Germany). Retinal explants were coupled in place with a weighted dialysis membrane, and continuously perfused with carboxygenated aCSF at 2.2 ml per minute using a peristaltic pump (SCI400, Watson Marlow, UK), and maintained at 32˚C using a Universal Serial Bus temperature controller regulating an inline heater for the inflow of aCSF. Light stimuli (white light) were presented by a customised light engine source (Lumencor, USA or Thorlab LEDs). At brightest intensity (ND0) LEDs were $1x10^{15}$ total photons/cm^2/s for Lumencore and $8x10^{14}$ total photons/cm^2/s for Thorlab LEDs. Arduino Due card (Italy) controlled by programmes written in LabVIEW (Version 8.6, National Instruments, TX, USA) was used to control stimulus duration and intensity by altering LED output and adjusting filter wheel containing neutral-density filters (ThorLabs, UK) which reduce the intensity by x10. Stimuli were delivered at 2-second pulses of light (20s inter-stimulus interval) for 20-30 repeats at ND0, ND1 (10x dimmer) and ND2 (100x dimmer). Data were sampled at 25 kHz during the acquisition of both spontaneous and evoked activity and recorded for off-line sorting using Offline Sorter (Plexon). After removing clear

artifacts common to all channels, principal component analyses were used to discriminate single units, identified as distinct clusters of spikes within the principal component space, with a clear refractory period in the interspike interval distribution. Spike-sorted, single-unit data were then further analysed using Neuroexplorer (Nex Technologies) and MATLAB R2010a (The Mathworks Inc.).

In-vivo electrophysiology

Recordings were performed on two groups of rd^1 mice: group 1 (n = 7), one eye injected with AAV2-CAG-*RHO* and the other with AAV2-CAG-GFP; and group 2 (n = 5), one eye injected with AAV2-grm6-*RHO* and the other with AAV2-grm6-GFP. Mice were anaesthetised with urethane (intraperitoneal injection 1.55g/kg; 20% w/v; Sigma Aldrich, Poole, UK), ketamine and xylazine (100mg/kg ketamine and 10mg/kg xylazine; intraperitoneally) or isofluorane (initial dose of 2-3% and maintenance dose of 0.6-1.0% administered via a nose cone; GM-4, Narishige, Japan). Animals were mounted in a stereotaxic frame (SR-15M; Narishige International Ltd, London, UK) and core body temperature was maintained at 37 °C via a homeothermic heat mat (Harvard Apparatus, Edenbridge, UK). Pupils were dilated with atropine and mineral oil (Sigma Aldrich) was applied to retain corneal moisture. A small craniotomy and durotomy (~1 mm^2) was performed directly above each lateral geniculate nucleus (LGN) using stereotaxic coordinates according to mouse atlas (Paxinos and Franklin, 2001; hole centre= bregma: −2.46 mm; midline: −2.8). A 32-channel electrode (NeuroNexus Technologies Inc., MI, USA) was introduced to each LGN in the centre of the hole (medial shank: -2.5 mm relative to midline; depth: −2.6 mm relative to brain surface at 18 degrees angle) for simultaneous recording from both LGNs. A second recording was performed where electrodes were re-positioned and advanced 250µm dorsally with respect to bregma (at -2.71mm). Following electrode insertion mice were dark adapted for 30 minutes to allow neuronal activity to stabilize. Data were acquired using a Recorder64 system (Plexon, TX, USA) with signal amplification by a 20x gain AC-coupled head stage (Plexon, TX) followed by preamplifier conditioning providing a total gain of 3500x. Data were high-pass (300Hz) filtered and time-stamped neural waveforms were digitized simultaneously from all channels at a rate of 40 kHz. Multiunit data was then stored for offline sorting and analysis as for the MEA data described above. To confirm the location of recording sites, the recording electrode was dipped in fluorescent dye (Cell Tracker CM-DiI; Invitrogen) prior to insertion into the brain. After in-vivo recordings, the mouse's brain was removed and post-fixed overnight in 4% paraformaldehyde, prior to cryoprotection for 24 hours in 30% sucrose. 100µm coronal sections were then cut using a sledge microtome, mounted onto glass slides and cover slipped using Vectashield (Vector Laboratories, Inc.).

Visual stimuli

Visual stimuli were provided by LEDs (Thorlab λmax: 410 nm) and delivered via fiber optic to purpose-made eye cones tightly positioned onto each eye to minimise any potential light leak. A National Instruments card (USB-6229) controlled by programmes written in LabVIEW (Version 8.6, National Instruments, TX, USA) was used to control stimulus duration and intensity by altering LED output and adjusting filter wheel containing neutral-density (ND) filters (ThorLabs, UK). At brightest intensity (ND0) LEDs gave a corneal irradiance of 47 W/m^2 or $4x10^{15}$ of effective flux for rod opsin; estimated retinal irradiance is $8x10^{13}$ log photons/cm^2/s based upon the method [S1]. Light was measured using a spectroradiometer (Bentham Instruments Ltd., UK or Cambridge Research Systems Ltd., UK), which measured the relative power in mW/cm^2 at

wavelengths between 350-700nm. The effective quantal flux (in photons/cm^2/s) for each opsin was then estimated by weighting spectral irradiance according to pigment spectral efficiency using the formula: effective photon flux = $\int P(\lambda).s(\lambda).l(\lambda)d\lambda$ where $P(\lambda)$ is spectral irradiance in photons/cm^2/s/nm; $s(\lambda)$ is pigment spectral sensitivity approximated by the Govardovskii visual template [S2]; and $l(\lambda)$ is mouse lens transmission as measured by Jacobs and Williams [S3].

Light flashes were delivered according to a light protocol consisting of 2 parts. Part 1 included flashes from darkness: 2s light ON, 20s light OFF with 10s offset between each eye. This paradigm was repeated at least 10x at each ND filter. Retinal irradiance ranged from $8x10^{11}$ photons/cm^2/s at ND2 to $8x10^{13}$ photons/cm^2/s at ND0. Part 2 of the light protocol involved recording in light adapted conditions where 5-second steps of light were applied to a steady background illumination at Michelson contrast of 96%. There was a 20-second inter-stimulus interval and a 10-second offset between two eyes. This paradigm was repeated ten times.
Naturalistic movies were presented with a digital mirror device projector (DLP® LightCommanderTM; Logic PD Inc.), whose intrinsic light engine had been replaced with our own multispectral LED light source containing four independently controlled LEDs (λ_{max} at 405nm, 455nm, 525nm and 630nm; Phlatlight PT-120 Series (Luminus Devices). Light from the LEDs was combined by a series of dichroic mirrors (ThorLabs), and directed onto the mirror device. The movie was presented using Python running PsychoPy Version 1.70.00 software. It featured mice moving around a behavioural arena including movement and looming of different sized objects (subtending visual angles ranging from 0.5° to 36°) at a range of orientations, speeds and contrasts (maximum Michelson contrast at 96%). The movie was presented at irradiance 0.81 W/m^2 with estimated retinal irradiance of $1x10^{13}$ rod equivalent photons/cm^2/s). The movie lacked differences in colour, and changes in irradiance across time were minimal (standard deviation of irradiance = 5.94%). Previous validations in *wild-type* mice have shown undetectable responses for presentations of de-focussed versions, indicating that most activity was elicited by changes in spatial patterns and object motion.

Behaviour
The modified light/dark box (dimensions: length=40cm width=40cm and height=30cm, open top) was made of Perspex and its walls painted white except for the two long sides of each arena, which were kept clear. Two identical infra-red lamps were placed centrally above each arena, to allow visualisation under dark conditions.
The visual stimuli were displayed from two 17-inch flat screen computer monitors (Acer V173b or Dell E173FP and ViewSonic matched for power by adjusting screen brightness) facing clear walls of each arena, using a DualHead2Go Digital Edition external multi-display adapter (Matrox Graphics Inc.). A variety of visual stimuli were generated using a custom written program and displayed on one monitor at a time. Stimuli included switching from 'black' (minimum brightness) to 'white', from steady grey to full screen flicker (Figure S4B to S4E) or square-wave gratings without an associated change in irradiance. The stimuli were presented when the mouse was in the middle of one half of the arena and the spatial frequency of gratings are reported for this viewing distance. Spatial frequency would become lower (by up to 2x) if the mouse moved towards the monitor and higher if it moved away. In addition a natural movie (colour rendition of an owl swooping in slow motion Figure S4F) was presented. Light intensity from 400-700nm was measured using a spectroradiometer (Bentham Instruments Ltd., UK or Cambridge Research Systems Ltd., UK). For full screen modulations, corneal irradiance for a

mouse looking directly at the screen was measured (for ease of comparison with other studies) by using a cosine diffuser light placed in the appropriate location in the arena.

Before the experimental period, mice were handled and habituated to their novel environment over 5 days, at the same time each day, by leaving them in the experimental box with their cagemates for 30 min. Following the habituation period, behaviour experiments were conducted over several weeks at the same time each day. Each group of mice was allowed to undergo only one testing condition per day. On each test day, mice were brought into the testing room in their home cages, allowed to accommodate to the testing room conditions for 30 minutes and then each mouse was tested individually. Mice were placed into the open field box (randomly to east or west half) and allowed to move freely between two arenas. All test trials were recorded under infra-red conditions through a camcorder fitted with an infra-red filter (λ=665nm). The box was thoroughly cleaned with 70% ethanol after each test trial and allowed to air-dry before next mouse was placed into the box.

A recording trial began after 3 minutes of habituation. Each trial run consisted of 5 minutes of control stimulus, following which a test stimulus was presented on a screen facing an arena that contained a mouse at this time point. The recorded trials were stored for off-line analysis using a video tracking software device (EthoVision® XT 10.1 Noldus, Tracksys Ltd., UK). We analysed distance travelled by each mouse in the entire box and outputted results in 30 second bins. The mouse's ability to detect the visual stimuli was assessed as a change in distance travelled in the 30s either side of test stimulus appearance. As we had no strong *a priori* expectation that stimuli would increase vs. decrease activity, we used a two-tailed paired *t* test to detect changes in locomotion. To account for habituation to the novel stimulus, we tested for statistically significant response across the group of treated mice in a single presentation for most tests here. The exception was the drifting grating, in which we explored the robustness of responses over repeated presentations in *wild-type* and rd^1 (treated and control) mice. We found that responses were retained over multiple presentations, but that high repeat numbers did not necessarily maximise the likelihood of detecting small effect sizes, as mouse behaviour appeared to change as they became increasingly accustomed to the task. Thus, e.g. we found that baseline activity progressively increased in both rd^1-grm6-*RHO* and rd^1-grm6-GFP animals and the magnitude of increases in activity decreased beyond 10 repeats (data not shown).

Supplemental references

S1. Lyubarsky, A.L., Daniele, L.L., and Pugh, E.N., Jr. (2004). From candelas to photoisomerizations in the mouse eye by rhodopsin bleaching in situ and the light-rearing dependence of the major components of the mouse ERG. Vision Res *44,* 3235-3251.

S2. Govardovskii, V.I., Fyhrquist, N., Reuter, T., Kuzmin, D.G., and Donner, K. (2000). In search of the visual pigment template. Vis Neurosci *17,* 509-528.

S3. Jacobs, G.H., and Williams, G.A. (2007). Contributions of the mouse UV photopigment to the ERG and to vision. Doc Ophthalmol *115,* 137-144.
