# Supplementary material for: Restoration of Vision with Ectopic Expression of Human Rod Opsin
Source: Curr Biol. 2015 Aug 17;25(16):2111–22. doi: 10.1016/j.cub.2015.07.029 (PMC4540256; doi:10.1016/j.cub.2015.07.029)
Supplement: Document S2. Article plus Supplemental Information [file mmc2.pdf]

Article

Current Biology

Restoration of Vision with Ectopic Expression of Human Rod Opsin

Highlights

- Ectopic human rod opsin restores visual functions in advanced retinal degeneration
- Rod opsin has greater sensitivity than current optogenetic strategies
- Rod opsin-treated animals respond to spatial stimuli, flicker, and natural scenes
- As a human protein ordinarily found in retinal tissue, barriers to clinic are minimized

Authors

Jasmina Cehajic-Kapetanovic, Cyril Eleftheriou, Annette E. Allen, ..., Katherine E. Davis, Paul N. Bishop, Robert J. Lucas

Correspondence

paul.bishop@manchester.ac.uk (P.N.B.),
robert.lucas@manchester.ac.uk (R.J.L.)

In Brief

Cehajic-Kapetanovic at al. show that ectopically expressed human rod opsin restores vision in a mouse model of advanced retinal degeneration. The quality of the restored vision compares favorably, especially in terms of sensitivity, with alternative approaches, and using a native human protein reduces barriers to future clinical trials.

Cehajic-Kapetanovic et al., 2015, Current Biology *25*, 2111–2122
August 17, 2015 ©2015 The Authors
http://dx.doi.org/10.1016/j.cub.2015.07.029

Current Biology
Article

CellPress

Restoration of Vision with Ectopic Expression of Human Rod Opsin

Jasmina Cehajic-Kapetanovic,[1,2] Cyril Eleftheriou,[3] Annette E. Allen,[3] Nina Milosavljevic,[3] Abigail Pienaar,[3] Robert Bedford,[3] Katherine E. Davis,[3] Paul N. Bishop,[1,2,*] and Robert J. Lucas[3,*]
[1]Centre for Ophthalmology and Vision Sciences, Institute of Human Development, University of Manchester, Manchester M13 9PT, UK
[2]Manchester Royal Eye Hospital, CMFT, Manchester Academic Health Sciences Centre, Manchester M13 9NT, UK
[3]Faculty of Life Sciences, University of Manchester, Oxford Road, Manchester M13 9PT, UK
*Correspondence: paul.bishop@manchester.ac.uk (P.N.B.), robert.lucas@manchester.ac.uk (R.J.L.)
http://dx.doi.org/10.1016/j.cub.2015.07.029
This is an open access article under the CC BY license (http://creativecommons.org/licenses/by/4.0/).

SUMMARY

Many retinal dystrophies result in photoreceptor loss, but the inner retinal neurons can survive, making them potentially amenable to emerging optogenetic therapies. Here, we show that ectopically expressed human rod opsin, driven by either a non-selective or ON-bipolar cell-specific promoter, can function outside native photoreceptors and restore visual function in a mouse model of advanced retinal degeneration. Electrophysiological recordings from retinal explants and the visual thalamus revealed changes in firing (increases and decreases) induced by simple light pulses, luminance increases, and naturalistic movies in treated mice. These responses could be elicited at light intensities within the physiological range and substantially below those required by other optogenetic strategies. Mice with rod opsin expression driven by the ON-bipolar specific promoter displayed behavioral responses to increases in luminance, flicker, coarse spatial patterns, and elements of a natural movie at levels of contrast and illuminance (≈50–100 lux) typical of natural indoor environments. These data reveal that virally mediated ectopic expression of human rod opsin can restore vision under natural viewing conditions and at moderate light intensities. Given the inherent advantages in employing a human protein, the simplicity of this intervention, and the quality of vision restored, we suggest that rod opsin merits consideration as an optogenetic actuator for treating patients with advanced retinal degeneration.

INTRODUCTION

Inherited retinal degenerations (retinal dystrophies), such as retinitis pigmentosa, affect 1:2,500 people worldwide. Irrespective of etiology, most affect the outer retina and lead to progressive and permanent loss of photoreception. Severe visual impairment is common in advanced stages of the degeneration, and these conditions are currently incurable. However, despite the loss of outer retinal photoreceptors, inner retinal neurons, including bipolar and ganglion cells, can survive and retain their ability to send visual information to the brain [1, 2]. These neurons therefore, represent promising targets for emerging optogenetic therapies that aim to convert them into photoreceptors and recreate the photosensitivity that has been lost during degeneration [3].

Pioneering work has shown that electrophysiological responses to light can be restored to animal models of retinal degeneration by introducing a variety of optogenetic actuators to the surviving inner retina, including the mammalian photopigment melanopsin [4], prokaryotic photoactivated ion channels or pumps [5–10], synthetic light switches [11–14], and a synthetic photopigment (Opto-mGluR6) [15]. These interventions can also support behavioral light responses including, in some cases, maze navigation or optokinetic reflexes reliant upon detection of spatial patterns or fast temporal modulations (flicker). However, in most cases, these actuators function only under very bright light, and, to date, no clinically achievable optogenetic intervention has recreated spatiotemporal discrimination at commonly encountered light levels.

Here, we set out to determine whether it is possible to recreate vision in blind mice using ectopic expression of a natural human protein, rod opsin. Mammalian rod opsins are readily produced under heterologous expression and can couple to native signaling cascades in several cell types in a light-dependent manner [16–19]. We reasoned that if they did this also in neurons of the inner retina, they could restore photosensitivity, and that several features of this approach could be beneficial for clinical application. First, the use of a human protein, and indeed one ordinarily found in the retina, would minimize the potential for immunogenic adverse effects when applied to patients. Second, as a G protein-coupled receptor, rod opsin has access to mechanisms of signal amplification not available to directly light-gated ion channels and thus could have much higher light sensitivity. Finally, rod opsin has the potential to address the need for sensitivity normalization in vision. Detecting objects in our environment relies upon distinguishing local differences in relative luminance across the huge variation in background light intensity. That is only possible because photoreceptors adjust their sensitivity according to the background light intensity. Achieving that goal for optogenetic photoactivators is challenging, but ectopically expressed rod opsin could theoretically do so via two mechanisms. On the one hand, its G protein signaling cascade could show dynamic desensitization. On the

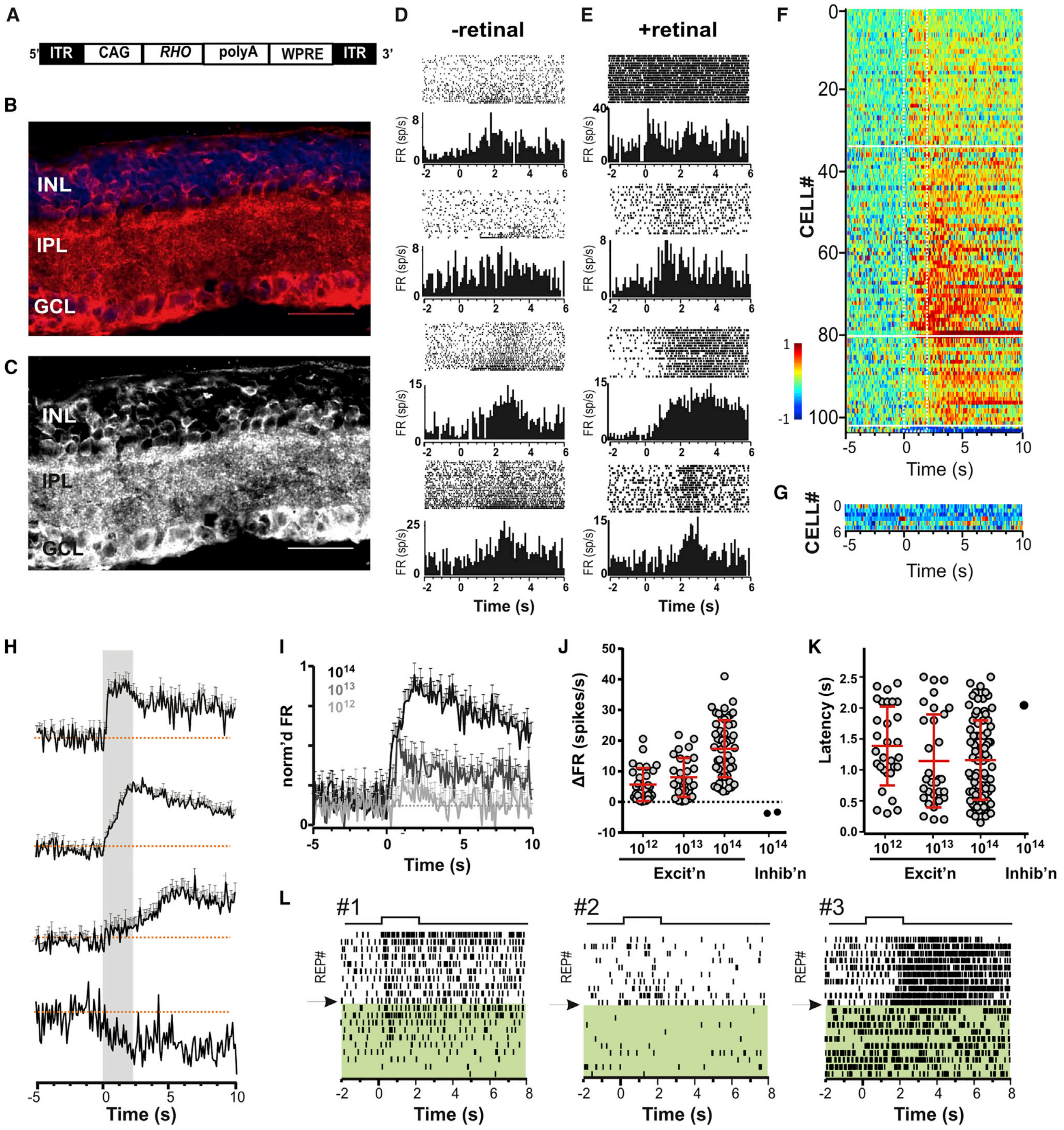

Figure 1. Ectopic Expression of Human Rod Opsin Restores Light Responses in *rd*[1] Mouse Retina

(A) Schematic of the DNA expression cassette delivered by AAV2/2 vector to the retina. A human rod opsin coding sequence (*RHO*) is driven by a hybrid CMV enhancer/chickenβ-actin (CAG) promoter. The sequence is flanked by inverted terminal repeats (ITRs) and stabilized by a polyadenylation signal sequence (polyA) and a woodchuck hepatitis posttranscriptional regulatory element (WPRE).

(B and C) Exemplar images of a section through an *rd*[1] mouse retina >4 months after intravitreal delivery of vector in (A) in conjunction with glycosidic enzymes. Expression of human rod opsin in cells of the ganglion cell layer (GCL) and inner nuclear layer (INL) and processes in the inner plexiform layer (IPL) are revealed by staining with an α-hRho antibody (red) and counterstaining of nuclei with DAPI (blue) to aid orientation (B). A monochrome version of α-hRho antibody staining in (B) in which rod opsin expression appears in white is shown in (C). Calibration bar = 50 μm.

(D and E) Perievent rasters and associated perievent firing rate histograms (PSTHs) for eight representative single units isolated from multi-electrode array (MEA) recordings of *rd*[1]-CAG-*RHO* retinas without (D) and with (E) exogenous 9-*cis*-retinal. Each set of rasters depicts spiking activity for 20 sequential presentations of a 2-s white light flash (4×10^{14} rod photons/cm^2/s; interstimulus interval 20 s) starting at time 0. PSTHs below depict mean firing rate in 100-ms epochs across all

(legend continued on next page)

other, because rod opsin bleaches upon light exposure, the effective concentration of pigment should be inversely proportional to the background irradiance. The associated reduction in sensitivity is well described for cone photoreceptors where it is termed “bleaching adaptation” [20, 21].

We expressed human rod opsin in surviving inner retinal neurons of a mouse model of aggressive retinal degeneration with near complete loss of rod and cone photoreceptors (*rd*[1]) by intravitreal administration of clinically approved adeno-associated virus (AAV) vector, AAV2/2. Widespread light-evoked changes in firing were observed in neurons of the retina and dorsal lateral geniculate nucleus (dLGN) in treated mice. These responses could be elicited using physiologically encountered light levels and under natural light-adapted conditions. Behavioral studies indicated that the treated mice had regained the ability to detect modest changes in brightness, relatively fast flickers, spatial patterns, and naturalistic movie scenes.

RESULTS

Gene Delivery to *rd*[1] Retina

We injected a viral vector (AAV2/2) containing a human rod opsin coding sequence under control of a CAG promoter (CAG-*RHO*; Figure 1A) into the vitreous of *rd*[1] mice in conjunction with glycosidic enzymes that increase vector transduction [22]. As predicted for this promoter, when retinas were harvested 4–6 months later, immunolabelling revealed rod opsin in both the ganglion cell layer (GCL) and inner nuclear layer (INL) of all treated *rd*[1] mice (Figures 1B and 1C). Expression was found at uneven density across the retina and was generally higher in GCL than INL (Figure S1A). Staining was absent from a control PBS-injected group (Figures S1B and S1C) and the inner retina of wild-type mice (Figures S1D and S1E). Patchy expression was also confirmed in retinal whole mounts for a reporter gene (GFP) delivered via a control AAV2-CAG-GFP vector (Figure S3A).

Restoring Light-Evoked Activity in Retinal Ganglion Cells

We tested for restored photosensitivity in CAG-*RHO*-transduced retinas by recording spiking activity from the GCL in vitro using a multi-electrode array. 2-s full-field flashes (interstimulus interval 20 s) of broad-spectrum white light increased spiking in numerous units (Figures 1D and 1E). Rod opsin bleaches upon light exposure, and, as might be expected, these responses dissipated over multiple repeats (Figure 1D) unless the culture medium was supplemented with 9-*cis*-retinal, when they became robustly repeatable (Figure 1E). We applied an objective criterion (see Experimental Procedures) to identify light-dependent changes in firing in these retinal explants. This returned 104 out of 671 single units as “light responsive” in CAG-*RHO*-treated retinas (Figure 1F) but only 6 out of 132 units in untreated *rd*[1] mice (Figure 1G). Closer examination of firing patterns in the six light-responsive units in control retinas provides little confidence that they did indeed respond to the stimulus, suggesting that these rather provide an indication of the false-positive rate of our objective test.

Restored ganglion cell light responses varied substantially in response latency (range 0.15 to 2.5 s at $\sim 4 \times 10^{14}$ rod-effective photons/cm^2/s) and amplitude (1.21 to 46.51 spikes/s at $\sim 4 \times 10^{14}$ rod-effective photons/cm^2/s; Figures 1H–1K). One-third of light-responsive units (n = 34) increased firing within 500 ms of the appearance of light, with a further 46 units responding between 500 ms and 1 s. However, longer delays were also observed (n = 24), including some units being excited after stimulus termination. A very small number of units decreased firing. Responses were obtained not only at maximum intensity ($\sim 4 \times 10^{14}$ rod-effective photons/cm^2/s) but also when irradiance was reduced by ×10 or ×100 (Figures 1I and 1J), with 31 and 30 units meeting our objective criterion of responsiveness at the two dimmer irradiances. This sensitivity is equivalent to that reported for Opto-mgluR6 [15] but superior to that of microbial photopigments and synthetic light switches, which generally require irradiances in the range 10^{14}–10^{17} photons/cm^2/s [5–14].

One interesting feature of restored light responses is that stimulus-induced increases in firing were much more numerous than decreases (Figure 1F). Rod opsin shows selectivity for $G\alpha_{i/o}$ class G proteins in heterologous expression [16–19], and one would therefore expect its primary light response to be inhibitory. Nevertheless, this could produce excitatory responses from retinal ganglion cells if it were to reduce the activity of inhibitory amacrine-cell synapses. Previous studies confirm that such sign inversions can occur in the degenerate retina [9, 15]. To test this possibility, we applied GABAa and GABAc receptor antagonists (TPMP 25 μM and picrotoxin 50 μM) to two retinal preparations. We found that excitatory responses were abolished by this treatment (Figure 1L, right-hand records) with the exception of one unit (Figure 1L, left-hand record). These data imply that the excitatory responses we observe originate primarily with light-dependent disinhibition of ganglion cell firing.

20 repeats. In both conditions, units show increases in firing associated with light presentation (from 0 to 2 s), but these are most pronounced for the first few trials (lower traces in raster) in (D), indicating bleaching, while inclusion of 9-*cis*-retinal (E) renders them repeatable across many trials.

(F and G) Heatmap representations of mean firing rate across at least 20 presentations of 2-s stimulus (ON at time 0) for 104 units from 5 *rd*[1]-CAG-*RHO* mice (F) and six units from three control *rd*[1]-CAG-GFP mice (G) meeting an objective criterion of stimulus-associated change in firing. Color code represents normalized firing rate (−1 and 1 being minimum and maximum firing rate for that unit, respectively). Traces are ordered according to response latency.

(H) Population mean (±SEM) normalized firing rate profiles for *rd*[1]-CAG-*RHO* units grouped according to response latency (horizontal white lines in F delineate extent of clusters).

(I) Mean ± SEM normalized firing rate (mean firing rate from −2 s to 6 s was normalized to maximum and minimum, and the normalized pre-stimulus firing rate (−2 to 0 s) was then subtracted) for all light-responsive units exposed to 2-s pulses (starting at 0 s) at 4×10^{14}, 4×10^{13}, and 4×10^{12} rod photons/cm^2/s.

(J and K) Distribution of response amplitudes (J; mean change in firing rate) and latencies (K; mean time at which mean firing rate first fell outside 2 SDs of baseline firing) for units in (F) responding with increases (excit’n) or decreases (inhib’n) in firing at 4×10^{14}, 4×10^{13}, and 4×10^{12} rod photons/cm^2/s.

(L) Perievent rasters for three single units showing firing of three units across multiple repeats of a 2-s light pulse (4×10^{14} rod photons/cm^2/s) without (above) and with (below; shaded in green) application of GABA receptor antagonists (TPMP 25 μM and picrotoxin 50 μM).

Characterization of Restored Responses In Vivo

To determine whether endogenous levels of *cis*-retinal in the degenerate retina were sufficient to allow ectopic rod opsin to function in vivo and how the signal recorded in the retina appeared in the brain, we turned to recording from the dLGN of anaesthetized mice using multi-electrode probes. For these experiments, we used animals in which one eye had been injected with the AAV2-CAG-*RHO* virus and the other with a control GFP virus (AAV2-CAG-GFP; Figure 2A). This enabled us to compare responses to stimuli presented to treated and control retinas in the same individual. We found that 2-s full-field flashes of 410-nm light (estimated retinal irradiance $\sim10^{14}$ rod photons/cm^2/s) produced many more responses when presented to the treated (Figure 2B) than controls (Figure 2C) eyes. In controls, we found 10 units (out of 736 single units in or around the dLGN) that met our objective criterion of light responsiveness. Several of these had very low baseline firing rate (Figure 2C), making them prone to appear as false positives according to our criterion of responsiveness, while the remainder had very sustained and/or delayed increases in firing as previously described for melanopsin-driven responses [23]. By contrast, stimuli presented to the treated eye induced changes in firing for 31 out of 736 units (Figure 2B). These could be either ipsi- or contra-lateral to the stimulated eye. Bleaching was not a problem for in vivo light responses, which showed robust firing across many repeated trials (Figure 2D) and even to light steps against a background (Figure 2E).

dLGN responses downstream from *rd*^{1}-CAG-*RHO* retinas were mostly excitatory in nature. Their response duration (0.56 ± 0.84 s; mean ± SD), amplitude, and latency were variable (Figures 2F and 2G), but a cluster of units responded within 500 ms of lights on. There were examples of cells that maintained elevated firing throughout light exposure, and in some cases beyond, while others showed more transient responses (Figure 2D). Responses could be discerned for stimuli at estimated retinal irradiance of 10^{14} and 10^{13}, but not 10^{12}, photons/cm^2/s (Figure 2D).

Restricting Ectopic Expression of Rod Opsin Using a Cell-Specific Promoter

A potential problem with untargeted expression of rod opsin is that the pigment will appear in cells that ordinarily would have quite different visual feature selectivity. This could make visual information in the brain incoherent. Therefore, we next selectively targeted rod opsin to ON-bipolar cells (Figure 3A) using an enhancer element derived from the grm6 promoter [24, 25] previously shown to drive expression in this cell type [6, 7, 8, 9, 12]. Viral transduction of a grm6-*RHO* construct resulted in rod opsin expression in cells of the INL across the retina (Figures S2A, S2B, and S2F) often clustered in patches of high transduction (Figure 3B; Figures S2C and S2D). Multi-electrode array recordings of the GCL of two grm6-*RHO*-treated retinas revealed stimulus-associated increases in firing in 30 out of 135 units (Figure 3C). Response latencies (Figure 3D; 1.14 ± 0.778 s; mean ± SD), durations (0.49 ± 0.76 s; mean ± SD), and amplitudes (Figure 3E; 2.8 ± 3.42 spikes/s; mean ± SD) varied significantly. Robust excitatory responses were observed at maximum light intensity ($\sim10^{14}$ rod photons/cm^2/s) and also when the intensity was reduced to $\sim10^{12}$ rod photons/cm^2/s (Figure 3F). Inhibition of GABAergic signaling abolished these responses (Figure 3G), consistent with the view that they arose primarily from a light-dependent disinhibition of ganglion cell firing.

Electrophysiological responses to light could be readily detected in the dLGN of grm6-*RHO*-treated animals. Thus, when presented with 2-s full-field flashes (410 nm; $\sim10^{14}$ rod equivalent photons/cm^2/s), numerous units (73 out of 481 units in or around the dLGN) showed a significant change in firing (Figure 3H). Once again, most responses were excitatory, but a number of inhibitory responses (n = 14) were also recorded in this case. Response latencies (Figure 3I; 1.07 ± 0.6; mean ± SD) and amplitudes (Figure 3J; 6.93 ± 9.377; mean ± SD) varied significantly, although many units responded within 500 ms of stimulus onset. Mean (±SD) response duration was 0.41(±0.28) s for increases and 1.21 (±0.75) s for decreases in firing. Responses were apparent at $\sim10^{14}$ and 10^{13} rod photons/cm^2/s but were less convincing when the stimulus intensity was dropped to 10^{12} rod photons/cm^2/s (Figure 3K).

Light-Induced Behavioral Responses

Next, we asked whether ectopic rod opsin could support visual discrimination. For this purpose, we set out to establish a behavioral test of vision that was higher throughput and less stressful than maze navigation tasks (which in our experience require very long training times for animals with poor vision [26]) and could be used in conjunction with a variety of visual features. Based upon previous light/dark box tests [7, 14, 27, 28] and other reports of behavioral responses to simple visual stimuli [29], we hypothesized that abrupt alterations in the visual scene might induce changes in spontaneous locomotor activity (either increases or decreases) that could be measured objectively with available image analysis software. Mice were placed in a modified light/dark box and allowed free movement between two arenas via an opening in the separating wall. Ordinary LCD computer monitors set to provide corneal irradiance 0.12 W/m^2 ($\sim$40 lux; retinal irradiance $\sim10^{11}$–10^{12} rod-equivalent photons/cm^2/s at maximum brightness “white screen” and a contrast ratio of 1:100) were placed behind transparent walls at either end of the arena. We started by asking whether mice could detect a simple luminance step by switching one of the monitors to “white” after the animals had been allowed to explore the box for several minutes with both monitors set to “black.” Wild-type mice responded to the change with an immediate increase in locomotor activity (Figure 4A). This response was absent from control *rd*^{1}-CAG-GFP mice, while both CAG-*RHO*- and grm6-*RHO*-treated mice responded to the appearance of the white screen with a statistically significant reduction in activity, indicating that they had detected the luminance increment (Figure 4A).

To probe temporal resolution of the restored vision, we investigated whether treated mice could detect the transition from a gray to a flickering screen of equivalent time-averaged irradiance (0.066 W/m^2). *rd*^{1}-grm6-*RHO* mice responded to appearance of either 2-Hz or 4-Hz flicker with decreased activity, while 10 Hz drove a significant increase (Figures 4B and 4C; two-way repeated measures [RM] ANOVA; $p < 0.0001$ for interaction between flicker frequency and gray versus flicker, post hoc Bonferroni correction $p < 0.05$ for gray versus flicker at 4 and 10 Hz; paired t test $p < 0.01$ also for 2 Hz). *rd*^{1}-CAG-*RHO* responded only to the 2-Hz flicker, while *rd*^{1}-CAG-GFP controls showed

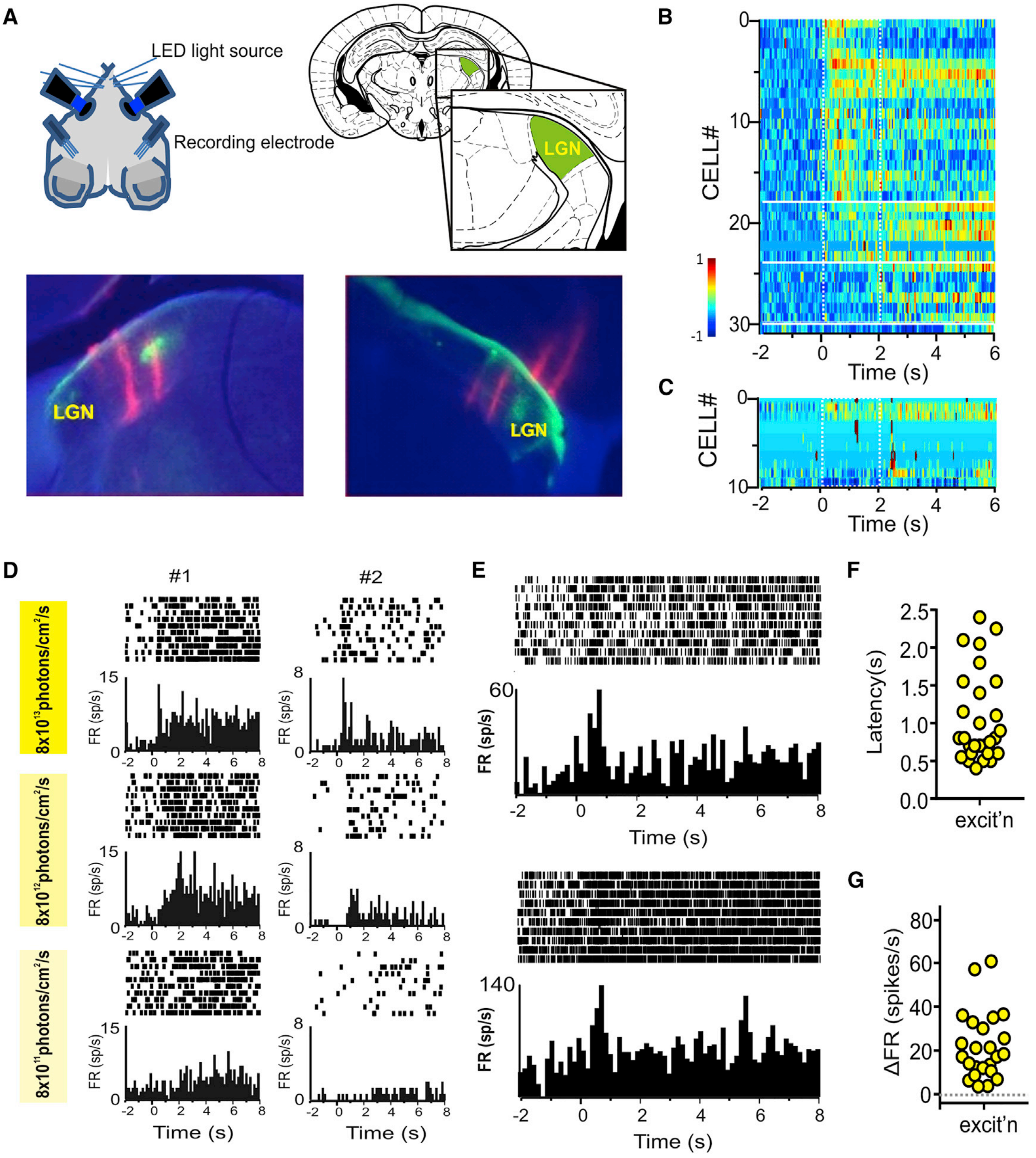

Figure 2. Rod Opsin Expression Driven by the Ubiquitous CAG Promoter Restores Light Responses in Blind rd^{1} Mouse Thalamus

(A) Schematic of recording apparatus allowing presentation of separate light stimuli to each eye and insertion of silicone multi-channel recording electrode probes to the dorsal lateral geniculate nuclei (dLGNs) in either hemisphere. Representative histological sections through the left and right dLGN with DiI tracks (in red) showing path of insertion for recording probes.

(B and C) Heatmap representations of mean firing rate across multiple presentations of 2-s stimulus (ON at time 0) to rd^{1}-CAG-*RHO* (B) and control rd^{1}-CAG-GFP (C) eyes of units showing a significant change in firing associated with stimulus presentation (n = 31 units downstream of 5 treated eyes and n = 10 units downstream of 5 control eyes). Color code represents normalized firing rate (−1 and 1 being minimum and maximum firing rate for that unit, respectively). Traces are ordered according to response latency.

(D) Sensitivity response profile (perievent rasters and associated perievent firing rate histograms) for two representative dLGN single units isolated from (B) at three different retinal irradiances: 8×10^{13}, 8×10^{12}, and 8×10^{11} rod-equivalent photons/cm^2/s.

(E) Light-adapted responses (perievent rasters and associated perievent firing rate histograms) for two representative dLGN units from rd^{1}-CAG-*RHO* eyes recorded under light-adapted conditions (retinal irradiance 8×10^{13} rod-equivalent photons/cm^2/s and Michelson contrast 96%).

(F and G) Distribution of response latencies (F; time at which mean firing rate first fell outside 2 SDs of baseline for units responding within 2.5 s of lights on) and amplitude (G; mean change in firing rate) for units in (B) responding with increases (excit'n) or decreases (inhib'n) in firing. CAG is a hybrid CMV enhancer/chickenβ-actin promoter. *RHO* is human rod opsin coding sequence.

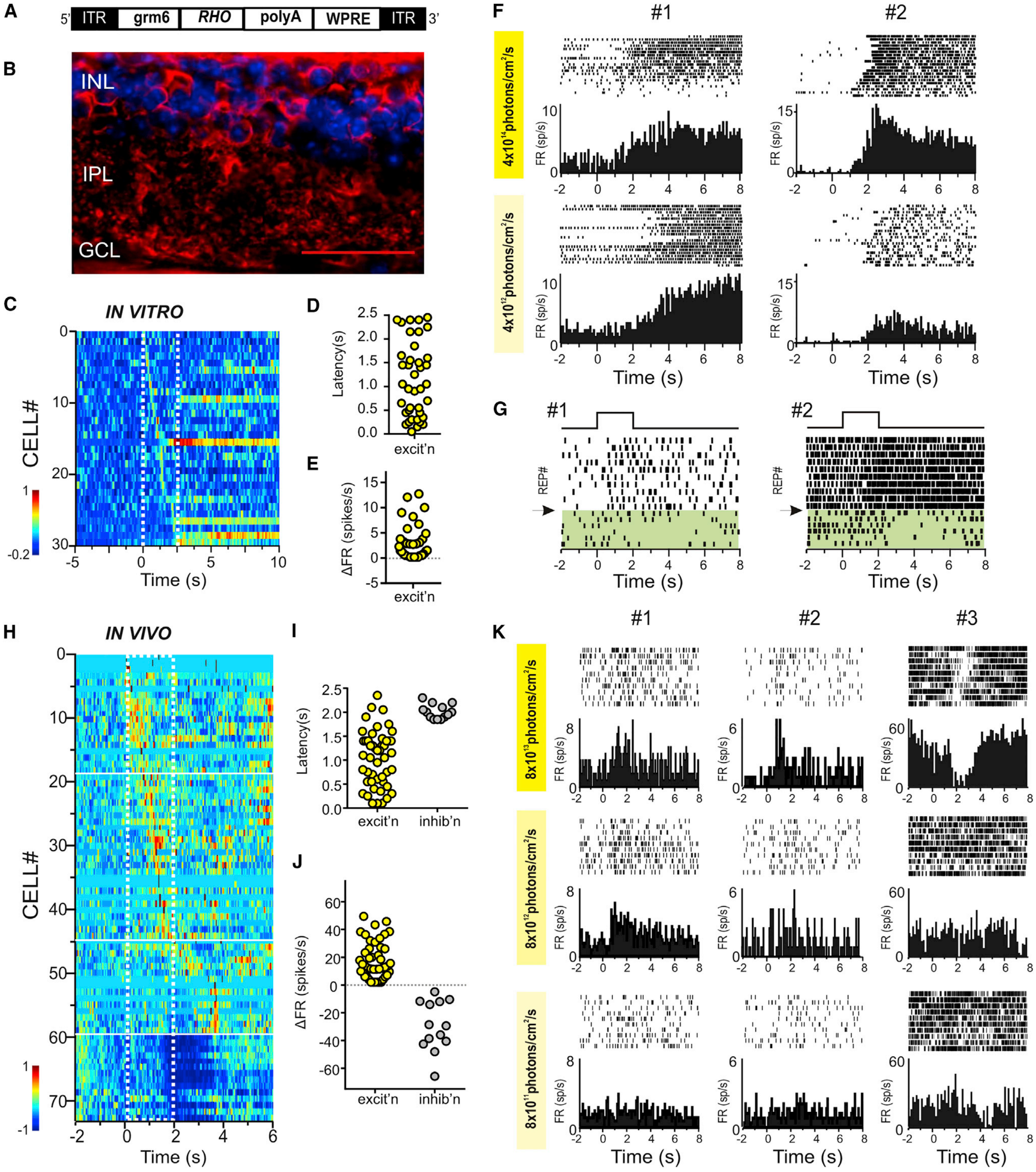

Figure 3. Selective Expression of Rod Opsin Using a Cell-Specific grm6 Promoter Restores Visual Responses in the dLGN of rd^1 Mice

(A) Schematic of the DNA expression cassette delivered by AAV2/2 vector to the retina, comprising *RHO* under the ON-bipolar cell-specific (grm6) promoter flanked by ITRs and stabilized by polyA and WPRE.

(B) Exemplar image of a section through an rd^1 mouse retina >4 months after intravitreal delivery of viral vector in (A) in conjunction with glycosidic enzymes. Expression of human rod opsin in cells of the INL and processes in the IPL are revealed by staining (red) with an α-hRho antibody and counterstaining of nuclei with DAPI (blue). Calibration bar = 50μm.

(legend continued on next page)

no behavioral response in this paradigm (Figure S4A). Using the 4-Hz flicker, we next explored the contrast sensitivity of the flicker detection by reducing the difference in brightness between white and black elements of the flicker (Figure 4D). We found that *rd1*-grm6-*RHO*-treated mice continued to respond when the contrast ratio was reduced from 1:100 to 1:50, but not 1:7 or lower (Figure 4D).

We used a different cohort of *rd1*-grm6-*RHO* mice to assess spatial acuity for the restored vision. In this case, we asked whether there was a change in locomotor activity associated with the switch from a uniform gray screen to a drifting grating (black:white contrast ratio = 1:7.5; stimuli matched for irradiance). We started by applying this paradigm to wild-type mice to confirm its suitability for our purpose. Appearance of these gratings induced increases in locomotor activity in wild-types at frequencies $\leq$0.4 or 0.6 cycles per degree (cpd) (Figure 4E, #1; first trial and Figure 4F average of seven trials; two-way RM ANOVA; $p < 0.01$ for gray versus gratings, post hoc Bonferroni correction $p < 0.05$ at 0.1 and 0.4 cpd; paired t test $p < 0.05$ also for 0.2 and 0.6 cpd). Importantly, this finding is consistent with published estimates of spatial acuity in mice from optokinetic and maze navigation methods [30, 31]. We tested treated mice first with a considerably lower grating frequency (0.04 cpd; equivalent to viewing 15-cm bars at 60-cm distance). We found that the grating induced an increase in activity in *rd1*-grm6-*RHO* mice (Figure 4E, #2 and #3). Across the population of treated mice, this approached statistical significance for the first single trial ($p = 0.05$) and was statistically significant ($p < 0.05$) over five (Figure 4G) or ten repeats ($p < 0.05$, data not shown). *rd1*-grm6-GFP mice showed no response to this stimulus (data not shown). When tested with a finer grating (0.08 cpd) neither *rd1*-grm6-*RHO* (Figure 4G) nor *rd1*-grm6-GFP (data not shown) mice showed a significant change in activity.

Visual Responses to Naturalistic Scenes

The ability of *rd1*-grm6-*RHO* to distinguish spatial patterns at contrast ratios (1:7.5) well within those experienced in natural scenes [32] led us to ask whether ectopic rod opsin might allow discrimination of more naturalistic scenes. We recorded electrophysiological activity in the dLGN across multiple repeats of a 30-s movie comprising mice moving around an open arena [33]. In both *rd1*-grm6-*RHO* and *rd1*-CAG-*RHO* mice, we found units whose firing rate appeared to increase at particular phases on multiple repeats of the movie, suggesting a response to features of the stimulus. However, only one of these from an *rd1*-grm6-*RHO* met an objective criterion of response (Figures 5A–5C). We finally asked whether treated mice could show behavioral responses to a natural movie by presenting a clip of a swooping owl (Figure 5D) to mice in the behavioral test arena. *rd1*-grm6-*RHO* mice responded to this stimulus with a significant increase in activity (Figures 5E and 5F), which was also observed in wild-type mice but was absent in control *rd1*-CAG-GFP mice or *rd1*-CAG-*RHO*-treated animals (Figure 5F).

DISCUSSION

We have found that ectopic expression of human rod opsin is an effective method of restoring vision in blind mice. Using electrophysiological recordings in the retina and visual thalamus, we find that ectopic rod opsin supports reproducible responses to light pulses and steps over a range of intensities typical of our everyday experience. At the single-unit level, restored responses can be excitatory or inhibitory, sustained or transient, mirroring the richness of the visual code seen in wild-type mice. Using a behavioral test, we find that rod opsin-treated mice are able to detect visual stimuli presented using an ordinary LCD visual display unit (VDU) in a dimly lit room. Under these conditions, they can distinguish flicker at a range of frequencies (up to 10 Hz), differences in luminance commonly encountered in visual scenes, coarse spatial patterns, and elements of a natural movie.

The quality of recreated vision reported here for human rod opsin has a number of encouraging characteristics and overall compares favorably with previous approaches. An important feature is its relatively high sensitivity. We find electrophysiological responses at retinal irradiances as low as $\sim 10^{12}$ photons/cm^2/s. This represents a significant improvement in sensitivity compared to previous studies using microbial opsins (thresholds between 10^{14} and 10^{17} photons/cm^2/s) [5–10], LiGluR/MAG photoswitches (10^{15}–10^{16} photons/cm^2/s) [11, 12], or photoactivated ligands (AAQ at 4×10^{15} photons/cm^2/s [13] and DENAQ at 4×10^{13} photons/cm^2/s) [14] and is similar to the most recent work with the synthetic Opto-mgluR6 receptor (6×10^{12} photons/cm^2/s) [15]. Importantly, this threshold for rod opsin-driven responses falls within the range of irradiances encountered in normal indoor environments.

(C) Heatmap representations of mean firing rate across multiple presentations of 2-s stimulus (ON at time 0) for 30 single retinal units from two *rd1*-grm6-*RHO* mice showing a significant change in firing associated with stimulus presentation. Color code represents normalized firing rate (−1 and 1 being minimum and maximum firing rate for that unit, respectively). Traces are ordered according to response latency.

(D and E) Distribution of response latencies (D; time at which mean firing rate fell outside 2 SDs of baseline for units responding within 2.5 s of lights on) and amplitude (E; mean change in firing rate) for units in (C) responding with increases (excit'n) in firing.

(F) Sensitivity response profile (perievent rasters and associated perievent firing rate histograms) for two representative retinal single units isolated from (C) at two different retinal irradiances: $4 \times 10^{14}$and 4×10^{12} rod-equivalent photons/cm^2/s.

(G) Perievent rasters for two single units showing inhibition of excitatory responses after application of GABA receptor antagonists (TPMP 25 μM and picrotoxin 50 μM; lower part of raster plots shaded in green).

(H) Heatmap representations of mean firing rate across multiple presentations of 2-s stimulus (ON at time 0) for 73 single dLGN units from *rd1*-grm6-*RHO* eyes showing a significant change in firing associated with stimulus presentation. Color code represents normalized firing rate (−1 and 1 being minimum and maximum firing rate for that unit, respectively). Traces are ordered according to response latency.

(I and J) Distribution of response latencies (I; time at which mean firing rate fell outside 2 SDs of baseline for units responding within 2.5 s of lights on) and amplitude (J; mean change in firing rate) for units in (C) responding with increases (excit'n) or decreases (inhib'n) in firing.

(K) Sensitivity response profile (perievent rasters and associated perievent firing rate histograms) for representative dLGN single units isolated from (H) at three different retinal irradiances: 8×10^{13}, 8×10^{12}, and 8×10^{11} rod-equivalent photons/cm^2/s.

CellPress

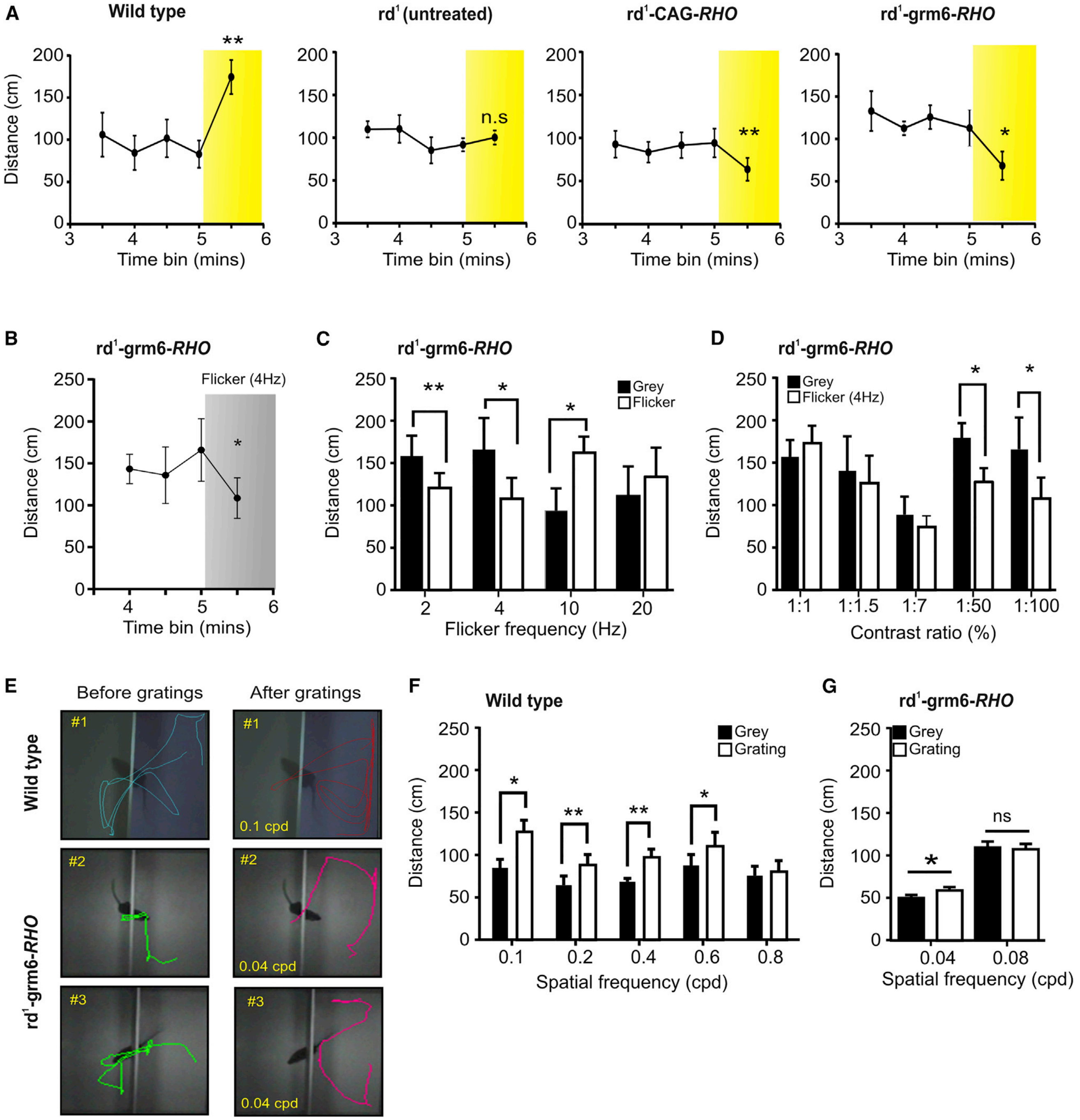

Figure 4. Ectopic Expression of Rod Opsin Restores Visual Behavior in Blind *rd1* Mice

(A) Open box activity plots for freely moving mice with LCD screens switched from "black" to "white" at time 5 min (illuminance 40 lux; estimated retinal irradiance 1×10^{12} rod-equivalent photons/cm^2/s).

(B) Open box activity plots for *rd1*-grm6-*RHO* mice exposed to 4-Hz flicker starting at 5 min (illuminance 20 lux; estimated retinal irradiance 8×10^{11} rod-equivalent photons/cm^2/s.

(C and D) Histograms of activity for *rd1*-grm6-*RHO* mice showing distance traveled in 30 s before (black bars) and 30 s after (white bars) presentation of "white" screen at different flicker frequencies (C) and at 4-Hz flicker at different contrast ratios (D).

(E) Representative movement trajectories for a wild-type and two different *rd1*-grm6-*RHO* mice in the open field box in the 30 s before (left) and 30 s after (right) presentation of gratings.

(F) Histogram of activity for wild-type mice showing distance traveled in 30 s before (black bars) and 30 s after (white bars) presentation of drifting squarewave gratings (contrast ratio 1:8) at different spatial frequencies.

(G) Histogram of change in activity in response to two different spatial frequencies (0.04 and 0.08 cpd) for *rd1*-grm6-*RHO* mice. Sample sizes for data in (A)–(D) are five wild-type, six *rd1*-CAG-GFP, six *rd1*-CAG-*RHO*, and five *rd1*-grm6-*RHO* mice; in (F) eight wild-type; in (G) nine *rd1*-grm6-*RHO*.

(legend continued on next page)

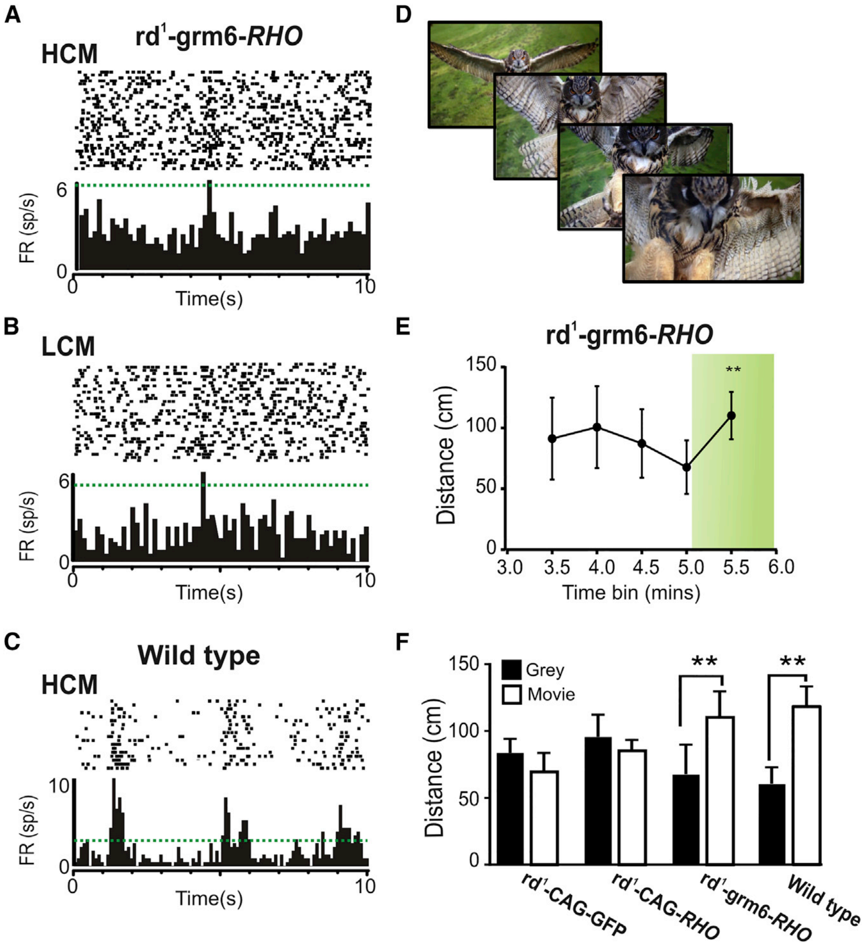

Figure 5. Rod Opsin Restores Visual Behavior in Response to Natural Scenes

(A and B) Perievent rasters and associated peri-event firing rate histograms for a dLGN unit to multiple presentations of a 30-s naturalistic movie (mice moving in an open arena in horizontal view; mean estimated retinal irradiance 1×10^{13} rod-equivalent photons/cm^2/s) to an *rd^1*-grm6-*RHO* eye. (A) and (B) show presentations of the high-contrast movie (HCM; black:white contrast ratio ≈ 1:100) and low-contrast movie (LCM; contrast ratio reduced 1:50), respectively. Horizontal line on histograms shows the 99% confidence interval for firing rate across the movie presentation; note the increase in firing above this line at the same time point for both movie presentations.

(C) Firing pattern of a representative dLGN unit from a wild-type mouse exposed to the HCM is presented for comparison.

(D) Example frames from a naturalistic movie featuring a swooping owl presented to mice in a behavioral arena.

(E) Open box activity plots for *rd^1*-grm6-*RHO* mice presented with a naturalistic swooping owl movie starting at 5 min (shaded in green; estimated retinal irradiance 8×10^{11} rod-equivalent photons/cm^2/s).

(F) Histogram of activity (mean ± SEM distance traveled by each animal) for *rd^1*-CAG-GFP (n = 6), *rd^1*-CAG-*RHO* (n = 6), *rd^1*-grm6-*RHO* (n = 5), and wild-type (n = 10) mice showing distance traveled in 30 s before (black bars) and after (white bars) presentation of the swooping owl movie. Two-tailed paired t tests comparing activity before and after stimulus appearance (**$p < 0.01$).

The relatively high sensitivity of the light responses driven by ectopic rod opsin raises the possibility that this intervention could allow visual discrimination under natural viewing conditions. We employed a new behavioral paradigm to determine the extent to which this was realized. Although developed independently, it is similar to a recently published approach shown to assay cortical vision [28]. At its heart is the prediction that an abrupt change in the visual scene may induce an alteration in behavioral state that can be measured as a change in locomotor activity. As commercially available software can measure mouse locomotor activity in open fields, we hoped that this would provide a simple and objective method to determine whether mice could distinguish between pairs of visual stimuli. That proved to be the case, and in wild-type mice, the new test replicates previous estimates of spatial acuity (Figure 4F) [30, 31]. When applied to treated animals, this behavioral test provides evidence for impressive visual discrimination in *rd^1*-grm6-*RHO* mice. These animals showed changes in activity not only to simple luminance increments but also to the appearance of more subtle visual cues including relatively fast flicker (up to 10 Hz) and simple spatial gratings.

Importantly, these responses were elicited under moderate illumination (∼20–150 lux; ∼10^{13} rod equivalent photons/cm^2/s) and at physiological levels of visual contrast. To our knowledge, this is the first time that a clinically amenable optogenetic intervention has been shown to support spatiotemporal discrimination under such natural viewing conditions. Optokinetic responses to drifting gratings have been recreated using both channelrhodopsin and halorhodopsin, but at much higher irradiances [8, 10]. In a recent study employing opto-mgluR6, such optokinetic responses were recorded at more physiological light levels [15]. However, that work was undertaken in a mouse line in which germline genetic modification was used to express the pigment in all ON-bipolar cells, confounding comparison with the effects of the more clinically relevant viral gene transfer employed here.

The behavioral responses of *rd^1*-grm6-*RHO* mice to relatively fast flicker (4 and 10 Hz) indicate that vision in these animals has

In all panels, activity is represented by mean ± SEM of the mean distance traveled by each animal in a 30-s time bin; time in min since introduction to testing arena. Two-tailed paired t tests comparing activity before and after stimulus appearance (*$p < 0.05$, **$p < 0.01$). For Figures 4B and 4C, two-way RM ANOVA; $p < 0.0001$ for interaction between flicker frequency and gray versus flicker, post hoc Bonferroni correction $p < 0.05$ for gray versus flicker at 4 and 10 Hz. For Figure 4F, two-way RM ANOVA; $p < 0.01$ for gray versus gratings, post hoc Bonferroni correction $p < 0.05$ at 0.1 and 0.4 cpd.

reasonable temporal resolution and that they can detect stimuli as short as 50 ms. However, it does not follow that they are able to actually resolve the flicker (i.e., detect the train of flashes) at these frequencies. Interactions with head and eye movements could produce apparent modulations at lower frequencies. Moreover, a temporal modulation in irradiance would also be apparent for a photoreceptor integrating over timescales that are not a perfect multiple of the flicker period (although note that the contrast of any such apparent temporal modulation would be strongly negatively correlated with integration period).

One potential advantage of rod opsin therapy is that it relies upon a light-absorbing chromophore (*cis*-retinal) that is naturally produced in the retina. A natural concern, however, is how the availability of the chromophore might be altered in retinal disease. On the one hand, degeneration of photoreceptors (which normally represent a substantial sink for chromophore) might make *cis*-retinal especially abundant in the surviving inner retina. On the other, secondary degeneration of the retinal pigment epithelium (RPE) can be a feature of advanced retinal degeneration, and some forms of dystrophy originate with visual-cycle defects. The effectiveness of rod opsin therapy in *rd1* mice (which exhibit RPE dystrophy [34]) argues that in many cases, the degenerate retina would contain sufficient chromophore. In other cases, augmentation with exogenous *cis*-retinal could be considered [35, 36].

In summary, the data presented here indicate that the level of vision recovered by ectopic expression of rod opsin compares favorably with that produced by other optogenetic actuators. Given the simplicity of the intervention and the inherent appeal of a therapy that entails introducing a human protein into a tissue in which it is ordinarily expressed, we suggest that human rod opsin warrants consideration as a method for restoring vision in advanced retinal degeneration.

EXPERIMENTAL PROCEDURES

See Supplemental Information for details on experimental procedures.

Adult C57BL/6J (wild-type) and C3H/HeJ (*rd1*) mice were used in this study. All animal experiments and care were conducted in accordance with the UK Animals (Scientific Procedures) Act (1986). Physiological and behavioral experiments were undertaken in mice between 8 and 12 weeks after intravitreal injection of AAV vector administered in isofluorane-anaesthetized mice between 8 and 10 weeks of age. Each eye was injected with 3 μl virus (1×10^{13} genomic counts) containing either a rod opsin (AAV2-ITR-CAG-*RHO*-polyA-WPRE-ITR for untargeted expression or AAV2-ITR-grm6-*RHO*-polyA-WPRE-ITR for targeted expression) or GFP (AAV2-ITR-CAG-GFP-polyA-WPRE-ITR for untargeted expression or AAV2-ITR-grm6-GFP-polyA-WPRE-ITR for targeted expression) expression construct, in combination with 0.5 μl of glycosidic enzyme solution containing 0.125 units each of heparinise III and hyaluronan lyase (E.C. 4.2.2.8 and E.C. 4.2.2.1; Sigma-Aldrich). Eyes were retrieved >6 weeks post vector injection, fixed, and cryosectioned before immunohistochemistry and microscopy. For details of gene delivery via AAV vector, histology, immunohistochemistry, and bio-imaging, see Supplemental Experimental Procedures.

Multi-electrode Array Recordings

Recordings were performed on rod opsin-treated *rd1* mice (n = 8) and GFP-injected *rd1* controls (n = 3) using a multi-electrode array system (Multi Channel Systems). Light stimuli (2-s full-field flashes of white light, 20-s interstimulus interval, at three different intensities 4×10^{12}, 4×10^{13}, and 4×10^{14} rod photons/cm^2/s) were presented by a customized light engine source (Lumencor or Thorlab LEDs). Spike-sorted, single-unit data were further analyzed using Neuroexplorer (Nex Technologies) and MATLAB R2010a (MathWorks).

In Vivo Electrophysiology

Lateral geniculate nucleus (LGN) recordings were performed on two groups of anaesthetized *rd1* mice using a 32-channel probe (Neuronexus). Group 1 (n = 7) had one eye injected with AAV2-CAG-*RHO* and the other with AAV2-CAG-GFP, and group 2 (n = 5) had one eye injected with AAV2-grm6-*RHO* and the other with AAV2-grm6-GFP. Visual stimuli were provided by LEDs (Thorlab λ_{max}: 410 nm) and delivered via fiber optic to purpose-made eye cones tightly positioned onto each eye to minimize any potential light leak. Light flashes were delivered according to a light protocol consisting of two parts. Part 1 included flashes from darkness: 2-s light ON, 20-s light OFF, with 10-s offset between each eye. This paradigm was repeated at least ten times at each neutral density (ND) filter. Retinal irradiance ranged from 8×10^{11} photons/cm^2/s at ND2 to 8×10^{13} photons/cm^2/s at ND0. Part 2 of the light protocol involved recording in light-adapted conditions where 5-s steps of light were applied to a steady background illumination at Michelson contrast of 96%. There was a 20-s interstimulus interval and a 10-s offset between two eyes. This paradigm was repeated ten times. Naturalistic movies were presented with a digital mirror device projector (DLP LightCommanderTM, Logic PD), whose intrinsic light engine had been replaced with our own multispectral LED light source containing four independently controlled LEDs (λ_{max} at 405 nm, 455 nm, 525 nm, and 630nm; Phlatlight PT-120 Series (Luminus Devices). For details, see Supplemental Information. We used the same objective criterion to identify light-responsive units in both in vitro and in vivo recordings—that firing rate within 4 s of the start of a 2-s pulse fell >2 SDs outside mean of baseline firing prior to light exposure. Applying this criterion to recordings from control *rd1* eyes provides confidence that it returns few false positives; the rate of false negatives is harder to determine. In addition to the responses shown here, it was our impression that in some cases, a light response appeared to have interacted with some underlying oscillatory mechanism, inducing a modest increase in firing around light stimulation and a more substantial change several seconds later. Response duration was estimated by the time over which firing rate fell outside 2 SDs of baseline. A few cells (n = 7 for CAG in vivo; n = 6 for grm6 in vitro; n = 7 for grm6 in vivo) in which the stimulus appeared to have induced a longer-lasting change in baseline firing patterns were not included in this analysis.

Behavior

Although developed independently, our test is similar to that in a recently published study [28] and shown by them to be a reflection of cortical vision. Using a modification of a light/dark box, mice were allowed free movement between two equal arenas (left and right halves) via an opening in the separating wall. The visual stimuli were displayed from two computer monitors (Acer V173b and either Dell E173FP or ViewSonic matched for power by adjusting screen brightness) facing clear walls of each arena, using a DualHead2Go Digital Edition external multi-display adaptor (Matrox Graphics). A variety of visual stimuli were generated using a custom-written program and displayed on one monitor at a time. For further details on behavioral set up and stimuli used, see Supplemental Information.

SUPPLEMENTAL INFORMATION

Supplemental Information includes Supplemental Experimental Procedures and four figures and can be found with this article online at http://dx.doi.org/10.1016/j.cub.2015.07.029.

AUTHOR CONTRIBUTIONS

J.C.-K., A.E.A., C.E., N.M., K.E.D., P.N.B., and R.J.L. designed the research. J.C.-K. performed intraocular injections, retinal histology, LGN recordings, and behavioral experiments. J.C.-K. and C.E. performed MEA recordings. A.P. performed behavioral experiments involving spatial stimuli with assistance from K.E.D., R.B., and N.M. J.C.-K. performed data processing and analysis with assistance from A.E.A. N.M. assisted with histology. J.C.-K., P.N.B., and R.J.L. wrote the manuscript with input from all authors. P.N.B. and R.J.L. supervised the research.

ACKNOWLEDGMENTS

We thank Franck Martial for assistance with light measurements for behavioral experiments; Jonathan Wynne and Dave Green for assistance with behavioral setup; and Timothy M. Brown for assistance with design and advice on electrophysiology experiments. J.C.-K. was supported by a Medical Research Council Clinical Research Training Fellowship (G1000268/1). This study was supported by grants from the ERC (268970 to R.J.L.), BBSRC (BB/K002252/1 to R.J.L.), and MRC (Confidence in Concept award MC_PC_13070 to R.J.L., P.N.B., and J.C.-K.).

Received: May 15, 2015
Revised: June 19, 2015
Accepted: July 10, 2015
Published: July 30, 2015

REFERENCES

1. Mazzoni, F., Novelli, E., and Strettoi, E. (2008). Retinal ganglion cells survive and maintain normal dendritic morphology in a mouse model of inherited photoreceptor degeneration. J. Neurosci. *28*, 14282–14292.
2. Santos, A., Humayun, M.S., de Juan, E., Jr., Greenburg, R.J., Marsh, M.J., Klock, I.B., and Milam, A.H. (1997). Preservation of the inner retina in retinitis pigmentosa. A morphometric analysis. Arch. Ophthalmol. *115*, 511–515.
3. Busskamp, V., Picaud, S., Sahel, J.A., and Roska, B. (2012). Optogenetic therapy for retinitis pigmentosa. Gene Ther. *19*, 169–175.
4. Lin, B., Koizumi, A., Tanaka, N., Panda, S., and Masland, R.H. (2008). Restoration of visual function in retinal degeneration mice by ectopic expression of melanopsin. Proc. Natl. Acad. Sci. USA *105*, 16009–16014.
5. Bi, A., Cui, J., Ma, Y.P., Olshevskaya, E., Pu, M., Dizhoor, A.M., and Pan, Z.H. (2006). Ectopic expression of a microbial-type rhodopsin restores visual responses in mice with photoreceptor degeneration. Neuron *50*, 23–33.
6. Doroudchi, M.M., Greenberg, K.P., Liu, J., Silka, K.A., Boyden, E.S., Lockridge, J.A., Arman, A.C., Janani, R., Boye, S.E., Boye, S.L., et al. (2011). Virally delivered channelrhodopsin-2 safely and effectively restores visual function in multiple mouse models of blindness. Mol. Ther. *19*, 1220–1229.
7. Cronin, T., Vandenberghe, L.H., Hantz, P., Juttner, J., Reimann, A., Kacsó, A.E., Huckfeldt, R.M., Busskamp, V., Kohler, H., Lagali, P.S., et al. (2014). Efficient transduction and optogenetic stimulation of retinal bipolar cells by a synthetic adeno-associated virus capsid and promoter. EMBO Mol. Med. *6*, 1175–1190.
8. Lagali, P.S., Balya, D., Awatramani, G.B., Münch, T.A., Kim, D.S., Busskamp, V., Cepko, C.L., and Roska, B. (2008). Light-activated channels targeted to ON bipolar cells restore visual function in retinal degeneration. Nat. Neurosci. *11*, 667–675.
9. Macé, E., Caplette, R., Marre, O., Sengupta, A., Chaffiol, A., Barbe, P., Desrosiers, M., Bamberg, E., Sahel, J.A., Picaud, S., et al. (2015). Targeting channelrhodopsin-2 to ON-bipolar cells with vitreally administered AAV Restores ON and OFF visual responses in blind mice. Mol. Ther. *23*, 7–16.
10. Busskamp, V., Duebel, J., Balya, D., Fradot, M., Viney, T.J., Siegert, S., Groner, A.C., Cabuy, E., Forster, V., Seeliger, M., et al. (2010). Genetic reactivation of cone photoreceptors restores visual responses in retinitis pigmentosa. Science *329*, 413–417.
11. Caporale, N., Kolstad, K.D., Lee, T., Tochitsky, I., Dalkara, D., Trauner, D., Kramer, R., Dan, Y., Isacoff, E.Y., and Flannery, J.G. (2011). LiGluR restores visual responses in rodent models of inherited blindness. Mol. Ther. *19*, 1212–1219.
12. Gaub, B.M., Berry, M.H., Holt, A.E., Reiner, A., Kienzler, M.A., Dolgova, N., Nikonov, S., Aguirre, G.D., Beltran, W.A., Flannery, J.G., and Isacoff, E.Y. (2014). Restoration of visual function by expression of a light-gated mammalian ion channel in retinal ganglion cells or ON-bipolar cells. Proc. Natl. Acad. Sci. USA *111*, E5574–E5583.
13. Polosukhina, A., Litt, J., Tochitsky, I., Nemargut, J., Sychev, Y., De Kouchkovsky, I., Huang, T., Borges, K., Trauner, D., Van Gelder, R.N., and Kramer, R.H. (2012). Photochemical restoration of visual responses in blind mice. Neuron *75*, 271–282.
14. Tochitsky, I., Polosukhina, A., Degtyar, V.E., Gallerani, N., Smith, C.M., Friedman, A., Van Gelder, R.N., Trauner, D., Kaufer, D., and Kramer, R.H. (2014). Restoring visual function to blind mice with a photoswitch that exploits electrophysiological remodeling of retinal ganglion cells. Neuron *81*, 800–813.
15. van Wyk, M., Pielecka-Fortuna, J., Löwel, S., and Kleinlogel, S. (2015). Restoring the ON switch in blind retinas: Opto-mGluR6, a next-generation, cell-tailored optogenetic tool. PLoS Biol. *13*, e1002143.
16. Li, X., Gutierrez, D.V., Hanson, M.G., Han, J., Mark, M.D., Chiel, H., Hegemann, P., Landmesser, L.T., and Herlitze, S. (2005). Fast noninvasive activation and inhibition of neural and network activity by vertebrate rhodopsin and green algae channelrhodopsin. Proc. Natl. Acad. Sci. USA *102*, 17816–17821.
17. Gutierrez, D.V., Mark, M.D., Masseck, O., Maejima, T., Kuckelsberg, D., Hyde, R.A., Krause, M., Kruse, W., and Herlitze, S. (2011). Optogenetic control of motor coordination by Gi/o protein-coupled vertebrate rhodopsin in cerebellar Purkinje cells. J. Biol. Chem. *286*, 25848–25858.
18. Cao, P., Sun, W., Kramp, K., Zheng, M., Salom, D., Jastrzebska, B., Jin, H., Palczewski, K., and Feng, Z. (2012). Light-sensitive coupling of rhodopsin and melanopsin to G(i/o) and G(q) signal transduction in Caenorhabditis elegans. FASEB J. *26*, 480–491.
19. Bailes, H.J., and Lucas, R.J. (2013). Human melanopsin forms a pigment maximally sensitive to blue light (λmax ≈ 479 nm) supporting activation of G(q/11) and G(i/o) signalling cascades. Proc. Biol. Sci. *280*, 20122987.
20. Pugh, E.N., Jr., Nikonov, S., and Lamb, T.D. (1999). Molecular mechanisms of vertebrate photoreceptor light adaptation. Curr. Opin. Neurobiol. *9*, 410–418.
21. Perlman, I., and Normann, R.A. (1998). Light adaptation and sensitivity controlling mechanisms in vertebrate photoreceptors. Prog. Retin. Eye Res. *17*, 523–563.
22. Cehajic-Kapetanovic, J., Le Goff, M.M., Allen, A., Lucas, R.J., and Bishop, P.N. (2011). Glycosidic enzymes enhance retinal transduction following intravitreal delivery of AAV2. Mol. Vis. *17*, 1771–1783.
23. Brown, T.M., Gias, C., Hatori, M., Keding, S.R., Semo, M., Coffey, P.J., Gigg, J., Piggins, H.D., Panda, S., and Lucas, R.J. (2010). Melanopsin contributions to irradiance coding in the thalamo-cortical visual system. PLoS Biol. *8*, e1000558.
24. Masu, M., Iwakabe, H., Tagawa, Y., Miyoshi, T., Yamashita, M., Fukuda, Y., Sasaki, H., Hiroi, K., Nakamura, Y., Shigemoto, R., et al. (1995). Specific deficit of the ON response in visual transmission by targeted disruption of the mGluR6 gene. Cell *80*, 757–765.
25. Kim, D.S., Matsuda, T., and Cepko, C.L. (2008). A core paired-type and POU homeodomain-containing transcription factor program drives retinal bipolar cell gene expression. J. Neurosci. *28*, 7748–7764.
26. Brown, T.M., Tsujimura, S., Allen, A.E., Wynne, J., Bedford, R., Vickery, G., Vugler, A., and Lucas, R.J. (2012). Melanopsin-based brightness discrimination in mice and humans. Curr. Biol. *22*, 1134–1141.
27. Bourin, M., and Hascoët, M. (2003). The mouse light/dark box test. Eur. J. Pharmacol. *463*, 55–65.
28. Cooke, S.F., Komorowski, R.W., Kaplan, E.S., Gavornik, J.P., and Bear, M.F. (2015). Visual recognition memory, manifested as long-term habituation, requires synaptic plasticity in V1. Nat. Neurosci. *18*, 262–271.
29. Yilmaz, M., and Meister, M. (2013). Rapid innate defensive responses of mice to looming visual stimuli. Curr. Biol. *23*, 2011–2015.
30. Gianfranceschi, L., Fiorentini, A., and Maffei, L. (1999). Behavioural visual acuity of wild type and bcl2 transgenic mouse. Vision Res. *39*, 569–574.
31. Prusky, G.T., West, P.W., and Douglas, R.M. (2000). Behavioral assessment of visual acuity in mice and rats. Vision Res. *40*, 2201–2209.

32. Mante, V., Frazor, R.A., Bonin, V., Geisler, W.S., and Carandini, M. (2005). Independence of luminance and contrast in natural scenes and in the early visual system. Nat. Neurosci. *8*, 1690–1697.

33. Allen, A.E., Storchi, R., Martial, F.P., Petersen, R.S., Montemurro, M.A., Brown, T.M., and Lucas, R.J. (2014). Melanopsin-driven light adaptation in mouse vision. Curr. Biol. *24*, 2481–2490.

34. Neuhardt, T., May, C.A., Wilsch, C., Eichhorn, M., and Lütjen-Drecoll, E. (1999). Morphological changes of retinal pigment epithelium and choroid in rd-mice. Exp. Eye Res. *68*, 75–83.

35. Van Hooser, J.P., Aleman, T.S., He, Y.G., Cideciyan, A.V., Kuksa, V., Pittler, S.J., Stone, E.M., Jacobson, S.G., and Palczewski, K. (2000). Rapid restoration of visual pigment and function with oral retinoid in a mouse model of childhood blindness. Proc. Natl. Acad. Sci. USA *97*, 8623–8628.

36. Ablonczy, Z., Crouch, R.K., Goletz, P.W., Redmond, T.M., Knapp, D.R., Ma, J.X., and Rohrer, B. (2002). 11-cis-retinal reduces constitutive opsin phosphorylation and improves quantum catch in retinoid-deficient mouse rod photoreceptors. J. Biol. Chem. *277*, 40491–40498.

Current Biology

Supplemental Information

Restoration of Vision with Ectopic Expression of Human Rod Opsin

Jasmina Cehajic-Kapetanovic, Cyril Eleftheriou, Annette E. Allen, Nina Milosavljevic, Abigail Pienaar, Robert Bedford, Katherine E. Davis, Paul N. Bishop, and Robert J. Lucas

Figure S1

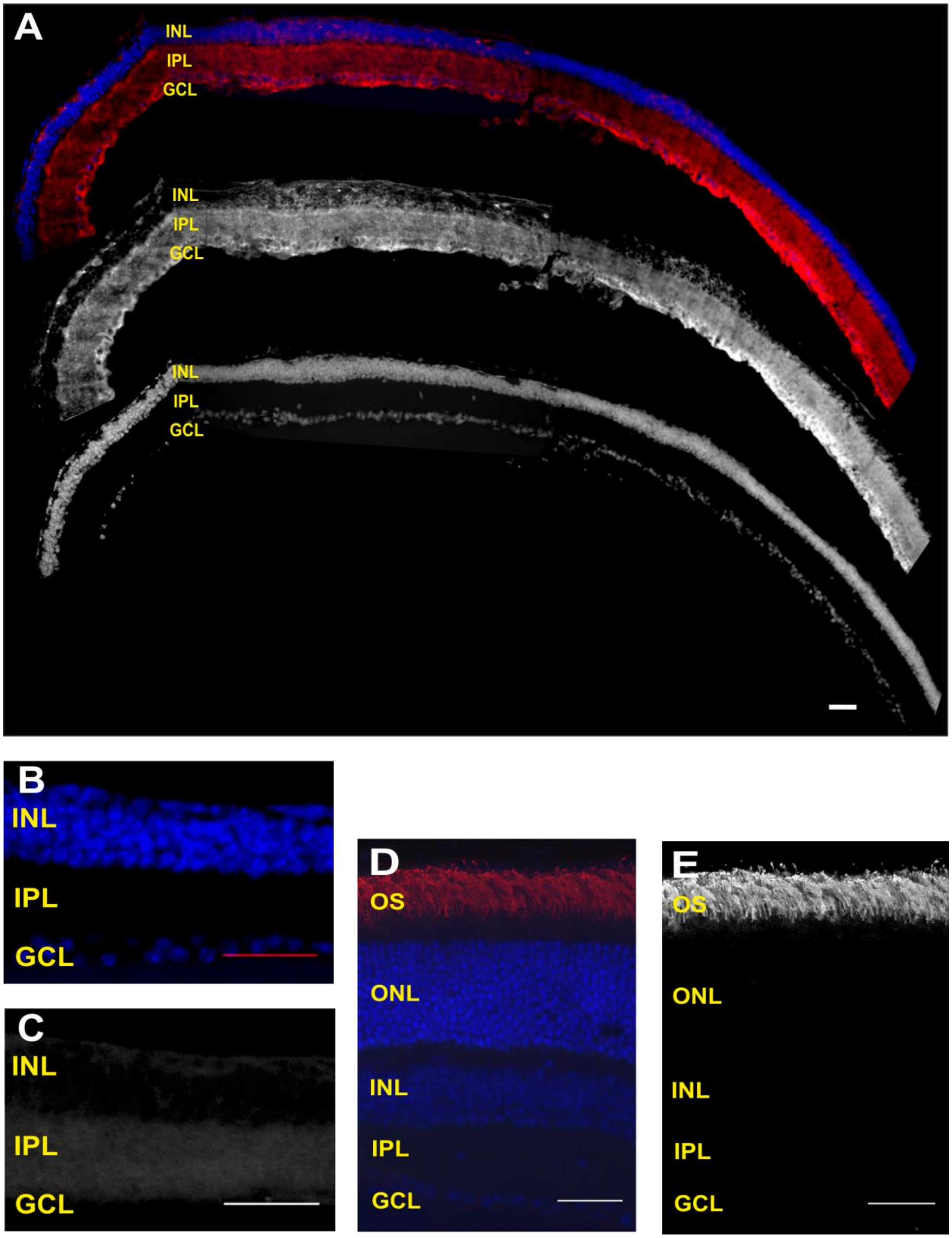
A
INL
IPL
GCL
INL
IPL
GCL
INL
IPL
GCL
B
INL
IPL
GCL
C
INL
IPL
GCL
D
OS
ONL
INL
IPL
GCL
E
OS
ONL
INL
IPL
GCL

Figure S2

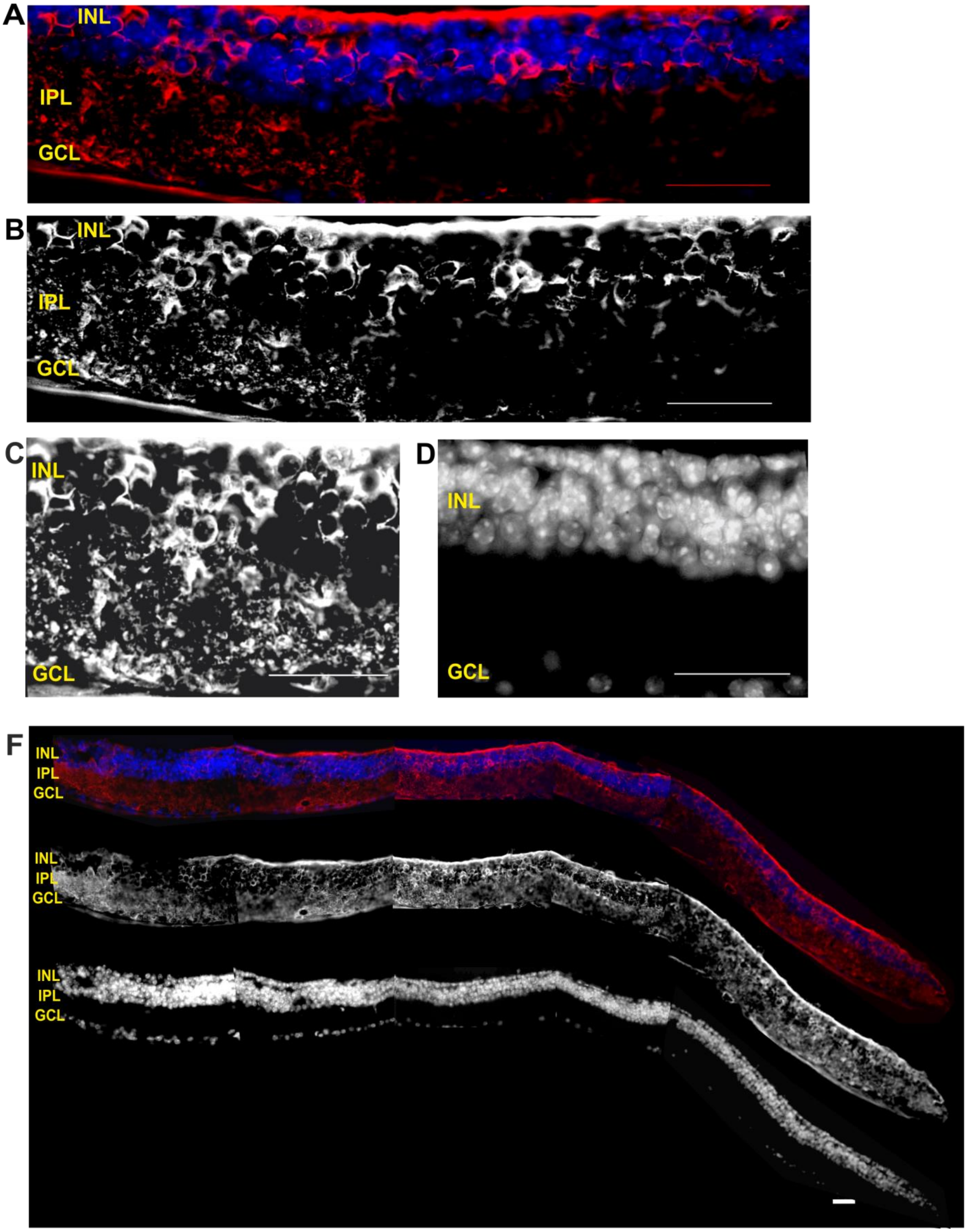
A
INL
IPL
GCL
B
INL
IPL
GCL
C
INL
GCL
D
INL
GCL
F
INL
IPL
GCL
INL
IPL
GCL
INL
IPL
GCL

Figure S3

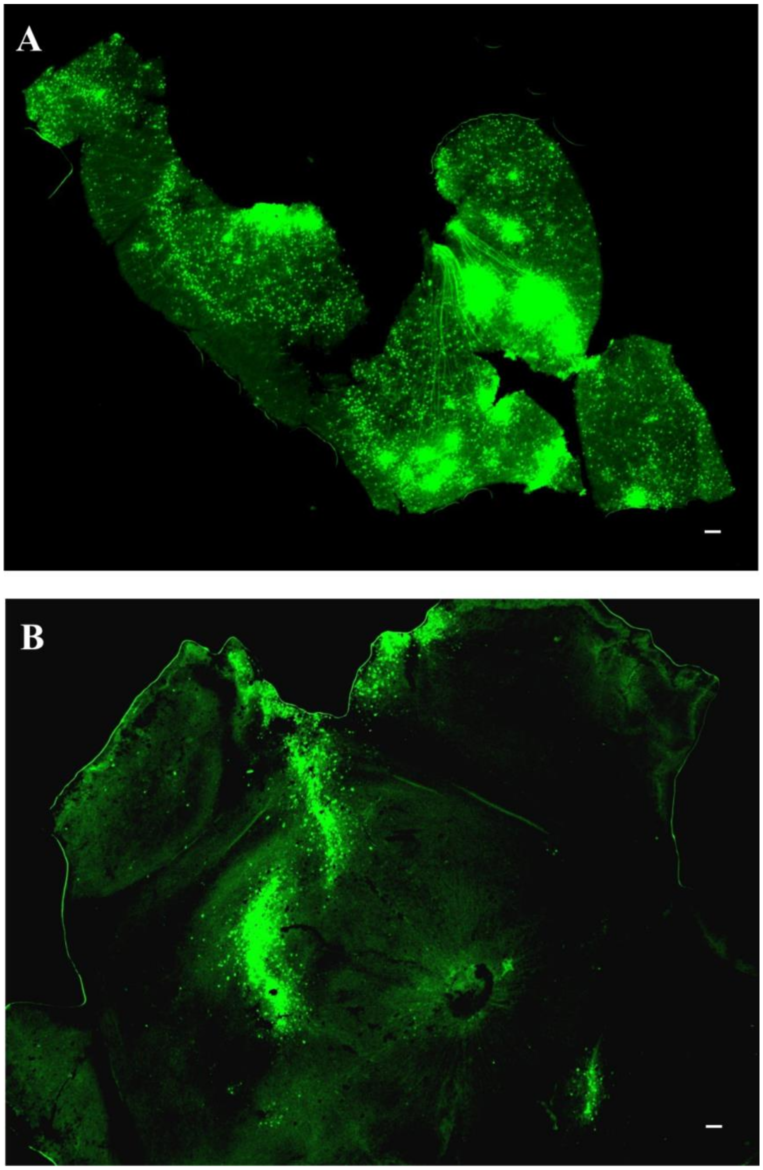

Figure S4

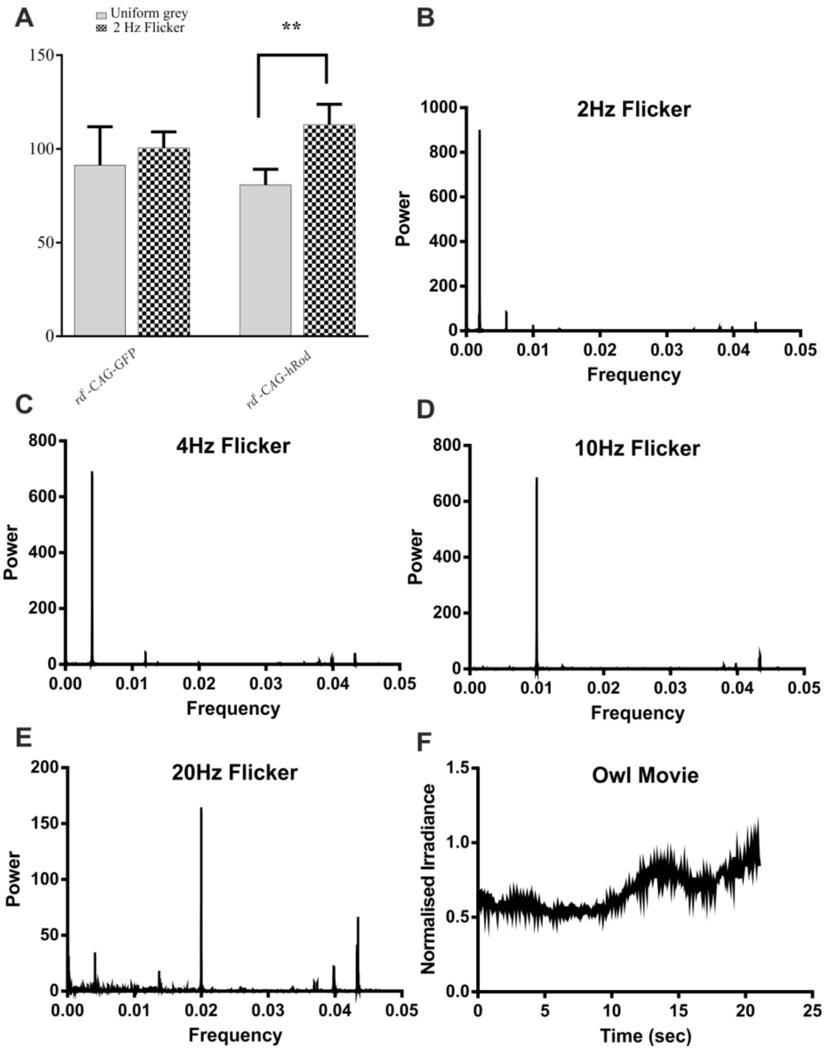
A
Uniform grey
2 Hz Flicker
**
150
100
50
0
rd1-CAG-GFP
rd1-CAG-hRod
B
2Hz Flicker
Power
Frequency
C
4Hz Flicker
D
10Hz Flicker
E
20Hz Flicker
F
Owl Movie
Normalised Irradiance
Time (sec)

Figure S1. Rod opsin expression in *rd^1* retinas using a non-selective (CAG) promoter and control *rd^1* and *wild-type* stains. Relating to Figure 1.
(A) Exemplar images of a section through an *rd^1* mouse retina >4 months after intravitreal delivery of AAV2/2-CAG-*RHO* in conjunction with glycosidic enzymes. Uneven density of rod opsin expression is shown after staining with α-hRho antibody (upper image in red; middle image in white, a monochrome version of antibody staining). Nuclei are stained with DAPI (upper image in blue; lower monochrome image in white). Note that this impression of staining across a retinal section was produced by splicing together several high magnification images of smaller portions of the retina. (B to E) images of sections through PBS injected *rd^1* (B and C) and *wild-type* (D and E) mouse retina showing no staining (B and C) and photoreceptor outer segment (OS) staining (D and E) after treatment with α-hRho antibody; nuclei are stained with DAPI (blue); C and E are monochrome versions of α-hRho antibody staining in (B) and (D) respectively; nuclei are stained with DAPI (blue). INL – inner nuclear layer, IPL- inner plexiform layer, GCL – ganglion cell layer, ONL – outer nuclear layer. CAG - a hybrid CMV enhancer/chickenβ-actin promoter. *RHO* - human rod opsin coding sequence. Calibration bar = 50µm.

Figure S2. Rod opsin expression in *rd^1* retina using an ON-bipolar specific (grm6) promoter. Relating to Figure 3.
(A) A longer section of the retina presented in Figure 3B >4 months after intravitreal delivery of AAV2/2-grm6-*RHO* in conjunction with glycosidic enzymes showing the extent of rod opsin expression in cells of the inner nuclear layer (INL) and processes in the inner plexiform layer (IPL) after staining (red) with an α-hRho antibody and counterstaining of nuclei with DAPI (blue). (B) A monochrome version of α-hRho antibody staining in (A) clearly depicting rod opsin expression in white. (C) A monochrome version of α-hRho antibody staining in Figure 3B showing a cluster of moderately high rod opsin expression (white). (D) A monochrome version of DAPI staining in Figure 3B. (F) Exemplar images of a section through an *rd^1* mouse retina >4 months after intravitreal delivery of AAV2/2-grm6-hRho in conjunction with glycosidic enzymes. Uneven density of rod opsin expression is shown after staining with α-hRho antibody (upper image in red) and (middle image in white, a monochrome version of antibody staining). Nuclei are stained with DAPI (upper image in blue and lower monochrome image in white). Note that this impression of staining across a retinal section was produced by splicing together several high magnification images of smaller portions of the retina. GCL - ganglion cell layer. grm6 - ON bipolar cell specific promoter. *RHO* - human rod opsin coding sequence. Calibration bar = 50µm.

Figure S3. GFP localisation in whole mount *rd^1* retinas following intravitreal injection of AAV2 driving transgene expression under a non-selective (CAG) promoter or an ON-bipolar specific (grm6) promoter. Relating to Figure 1 and Figure 3. Retinal wholemounts in both A (using a non-selective (CAG) promoter) and B (using an ON-bipolar specific (grm6) promoter) depict uneven expression of a marker (GFP) driven by these promoters. Transduced cells are shown in green. Calibration bar = 50µm.

Figure S4. Behavioural activity in treated and control *rd^1* mice in response to 2Hz flicker and visual stimuli used for behavioural experiments. Relating to Figure 4 and Figure 5. (A) Histogram of activity for *rd^1*-CAG-GFP (n = 6) and *rd^1*-CAG-*RHO* (n = 6) mice showing

distance travelled in an open field box 30s before (grey bars) and 30s after (chequered bars) presentation of a 2Hz full field flicker. Activity is represented by mean±SEM of the mean distance travelled by each animal in a 30-sec time bin; time in minutes since introduction to testing arena. Two tailed paired t-tests comparing activity before and after stimulus appearance (**$p < 0.01$). CAG - a hybrid CMV enhancer/chickenβ-actin promoter. *RHO* - human rod opsin coding sequence. (B-F) Flicker and naturalistic movie stimuli were generated using standard LCD monitors. To assess the characteristics of these stimuli we measured irradiance with a photodiode (Advanced Photonix SLD-70 BG2A) connected to a 10-bit analog-to-digital converter of a microcontroller board (Arduino UNO). The sampling rate of acquisition was 100 Hz. The power spectrum graphs show frequency in kHz and power in arbitrary unit. B-E show power spectrum analyses for irradiance traces at 2, 4, 10 and 20Hz. Note the contamination of lower frequency components at 20Hz (as we approach the limit of hardware performance) but not other frequencies. F shows the change in irradiance (normalised to max=1) over the course of a single repeat of the swooping owl movie.

Supplemental Experimental Procedures

Gene delivery via AAV

AAV vector was administered via intravitreal injection in isofluorane anaesthetised mice aged 8 to 10 weeks. Prior to injections, pupils were dilated with tropicamide and phenylephrine. A custom made ultra-fine needle (Hamilton RN needle 34 gauge, supplied by ESSLAB) was attached to a 5µl Hamilton glass syringe and was passed at 45 degrees through the pars plana into the vitreous cavity, carefully avoiding the lens and blood vessels. The injection was performed under a direct visualisation of the needle tip through cover-slipped eyes under an operating microscope (Microscopes Inc., USA). The vectors, rAAV serotype 2 (rAAV2/2, or simply AAV2) expressing rod opsin or GFP under the control of a strong ubiquitous pan-neuronal promoter (CAG) or ON-bipolar cell specific (grm6) promoter were obtained from Vector Biolabs, Philadelphia, USA. The CAG promoter is a fusion of CMV early enhancer and chicken β-actin promoter. The grm6 promoter is a fusion of 200-base pair enhancer sequence of the mouse grm6 gene encoding for ON-bipolar cell specific metabotropic glutamate receptor, mGluR6, and an SV40 eukaryotic promoter. The gene of interest in each case was flanked by inverted terminal repeat (ITR) domains and stabilised by polyadenylation signal sequence (polyA) and a woodchuck hepatitis posttranscriptional regulatory element (WPRE).

Each eye was injected with 3µl virus ($1x10^{13}$ genomic counts) containing either a rod opsin (AAV2-ITR-CAG-*RHO*-polyA-WPRE-ITR for untargeted expression or AAV2-ITR-grm6-*RHO*-polyA-WPRE-ITR for targeted expression) or GFP (AAV2-ITR-CAG-GFP-polyA-WPRE-ITR for untargeted expression or AAV2-ITR-grm6-GFP-polyA-WPRE-ITR for targeted expression) expression construct, in combination with 0.5µl of glycosidic enzyme solution containing 0.125 units each of heparinise III and hyaluronan lyase (E.C. 4.2.2.8 & E.C. 4.2.2.1; Sigma-Aldrich, Dorset, UK). The enzyme solutions were made fresh on the day of injection by dissolving the enzymes in sterile phosphate-buffered saline (PBS). The vector and enzymes were mixed in a syringe immediately before an eye injection and were given in a single combined injection.

Histology

Retrieved eyecups (>6 weeks post vector injection) were fixed in 4% paraformaldehyde (PFA) for 24 hours at 4° C. The tissue was then washed in PBS and further fixed in 30% sucrose in PBS overnight at 4° C. Fixed eyes were cryo-protected in optimal-cutting temperature medium (Raymond A Lamb Ltd., Eastbourne, UK) and frozen at -80° C until further processing. Cryo-protected retinal section were sectioned on a cryostat (Leica, Microsystems) horizontally through the eyecup at 8-10µm thickness from ventral to dorsal side, so that each section contained a complete nasal to temporal cross-section of the retina. Ten-twelve sections were collected on each slide containing sections representative of the entire retina. Slides were stored at -80° C.

For immunohistochemistry, slides were removed from the freezer and allowed to air-dry at room temperature for 1 hour. Sections were permeabilised by immersing slides in PBS with 0.2% Triton for 20 minutes at room temperature. Following this sections were background blocked with PBS with 0.2% Triton X-100 containing 10% donkey serum (D9663; Sigma, UK) for 1 hour at room temperature. Primary antibody (Rabbit Anti-Human Rhodopsin, Abcam, Ab112576) was applied at 1:200 dilution in blocking buffer (PBS with 0.2% Triton X-100 and 2.5% donkey serum) for 3 hours at room temperature. After washing in tween 0.05% PBS, four times for 10 minutes, sections were incubated with secondary antibody (Alexa Fluor® 546 Donkey Anti-Rabbit IgG (H+L) Antibody, Life technologies, lot: 1504518) diluted 1:200 in PBS with 0.2% Triton X-100 and 2.5% donkey serum for 2 hours at room temperature. Slides were then washed four times for 10 minutes in 0.05% tween PBS followed by one final wash with dH_20. After removing excess fluid, slides were mounted with fluorescent mounting media containing DAPI (Vectashield, Vector Laboratories Ltd., Peterborough, UK) to stain cell nuclei. For bio-imaging, sections were analysed under an Olympus BX51 upright microscope using x4, x10 and x20 Plan Fln objectives and captured using a Coolsnap ES camera (Photometrics, Tucson, AZ) through MetaVue Software (Molecular Devices Ltd. Wokingham, UK). Images were taken under specific band pass filter sets and colour-combined images were used for further processing using ImageJ.

Multi-electrode array recordings

Enucleated eyes were placed in a petri dish filled with carboxygenated (95% CO2/5%CO2) aCSF (artificial cerebro-spinal fluid, concentration in mM: 118 NaCl, 25 NaHCO3, 1 NaH2PO4, 3 KCl, 1 MgCl2, 2 CaCl2, 10 C6H12O6, 0.5 L-Glutamine). Retinas were then carefully isolated in diffuse red light under a dissecting microscope and mounted, ganglion cell side down, onto a 60- or 256-channel multi-electrode array (Multi Channel Systems, Reutlingen, Germany). Retinal explants were coupled in place with a weighted dialysis membrane, and continuously perfused with carboxygenated aCSF at 2.2 ml per minute using a peristaltic pump (SCI400, Watson Marlow, UK), and maintained at 32˚C using a Universal Serial Bus temperature controller regulating an inline heater for the inflow of aCSF. Light stimuli (white light) were presented by a customised light engine source (Lumencor, USA or Thorlab LEDs). At brightest intensity (ND0) LEDs were $1x10^{15}$ total photons/cm^2/s for Lumencore and $8x10^{14}$ total photons/cm^2/s for Thorlab LEDs. Arduino Due card (Italy) controlled by programmes written in LabVIEW (Version 8.6, National Instruments, TX, USA) was used to control stimulus duration and intensity by altering LED output and adjusting filter wheel containing neutral-density filters (ThorLabs, UK) which reduce the intensity by x10. Stimuli were delivered at 2-second pulses of light (20s inter-stimulus interval) for 20-30 repeats at ND0, ND1 (10x dimmer) and ND2 (100x dimmer). Data were sampled at 25 kHz during the acquisition of both spontaneous and evoked activity and recorded for off-line sorting using Offline Sorter (Plexon). After removing clear

artifacts common to all channels, principal component analyses were used to discriminate single units, identified as distinct clusters of spikes within the principal component space, with a clear refractory period in the interspike interval distribution. Spike-sorted, single-unit data were then further analysed using Neuroexplorer (Nex Technologies) and MATLAB R2010a (The Mathworks Inc.).

In-vivo electrophysiology

Recordings were performed on two groups of rd^1 mice: group 1 (n = 7), one eye injected with AAV2-CAG-*RHO* and the other with AAV2-CAG-GFP; and group 2 (n = 5), one eye injected with AAV2-grm6-*RHO* and the other with AAV2-grm6-GFP. Mice were anaesthetised with urethane (intraperitoneal injection 1.55g/kg; 20% w/v; Sigma Aldrich, Poole, UK), ketamine and xylazine (100mg/kg ketamine and 10mg/kg xylazine; intraperitoneally) or isofluorane (initial dose of 2-3% and maintenance dose of 0.6-1.0% administered via a nose cone; GM-4, Narishige, Japan). Animals were mounted in a stereotaxic frame (SR-15M; Narishige International Ltd, London, UK) and core body temperature was maintained at 37 °C via a homeothermic heat mat (Harvard Apparatus, Edenbridge, UK). Pupils were dilated with atropine and mineral oil (Sigma Aldrich) was applied to retain corneal moisture. A small craniotomy and durotomy (~1 mm^2) was performed directly above each lateral geniculate nucleus (LGN) using stereotaxic coordinates according to mouse atlas (Paxinos and Franklin, 2001; hole centre= bregma: −2.46 mm; midline: −2.8). A 32-channel electrode (NeuroNexus Technologies Inc., MI, USA) was introduced to each LGN in the centre of the hole (medial shank: -2.5 mm relative to midline; depth: −2.6 mm relative to brain surface at 18 degrees angle) for simultaneous recording from both LGNs. A second recording was performed where electrodes were re-positioned and advanced 250µm dorsally with respect to bregma (at -2.71mm). Following electrode insertion mice were dark adapted for 30 minutes to allow neuronal activity to stabilize. Data were acquired using a Recorder64 system (Plexon, TX, USA) with signal amplification by a 20x gain AC-coupled head stage (Plexon, TX) followed by preamplifier conditioning providing a total gain of 3500x. Data were high-pass (300Hz) filtered and time-stamped neural waveforms were digitized simultaneously from all channels at a rate of 40 kHz. Multiunit data was then stored for offline sorting and analysis as for the MEA data described above. To confirm the location of recording sites, the recording electrode was dipped in fluorescent dye (Cell Tracker CM-DiI; Invitrogen) prior to insertion into the brain. After in-vivo recordings, the mouse's brain was removed and post-fixed overnight in 4% paraformaldehyde, prior to cryoprotection for 24 hours in 30% sucrose. 100µm coronal sections were then cut using a sledge microtome, mounted onto glass slides and cover slipped using Vectashield (Vector Laboratories, Inc.).

Visual stimuli

Visual stimuli were provided by LEDs (Thorlab λmax: 410 nm) and delivered via fiber optic to purpose-made eye cones tightly positioned onto each eye to minimise any potential light leak. A National Instruments card (USB-6229) controlled by programmes written in LabVIEW (Version 8.6, National Instruments, TX, USA) was used to control stimulus duration and intensity by altering LED output and adjusting filter wheel containing neutral-density (ND) filters (ThorLabs, UK). At brightest intensity (ND0) LEDs gave a corneal irradiance of 47 W/m^2 or $4x10^{15}$ of effective flux for rod opsin; estimated retinal irradiance is $8x10^{13}$ log photons/cm^2/s based upon the method [S1]. Light was measured using a spectroradiometer (Bentham Instruments Ltd., UK or Cambridge Research Systems Ltd., UK), which measured the relative power in mW/cm^2 at

wavelengths between 350-700nm. The effective quantal flux (in photons/cm^2/s) for each opsin was then estimated by weighting spectral irradiance according to pigment spectral efficiency using the formula: effective photon flux = $\int P(\lambda).s(\lambda).l(\lambda)d\lambda$ where $P(\lambda)$ is spectral irradiance in photons/cm^2/s/nm; $s(\lambda)$ is pigment spectral sensitivity approximated by the Govardovskii visual template [S2]; and $l(\lambda)$ is mouse lens transmission as measured by Jacobs and Williams [S3].

Light flashes were delivered according to a light protocol consisting of 2 parts. Part 1 included flashes from darkness: 2s light ON, 20s light OFF with 10s offset between each eye. This paradigm was repeated at least 10x at each ND filter. Retinal irradiance ranged from $8x10^{11}$ photons/cm^2/s at ND2 to $8x10^{13}$ photons/cm^2/s at ND0. Part 2 of the light protocol involved recording in light adapted conditions where 5-second steps of light were applied to a steady background illumination at Michelson contrast of 96%. There was a 20-second inter-stimulus interval and a 10-second offset between two eyes. This paradigm was repeated ten times.
Naturalistic movies were presented with a digital mirror device projector (DLP® LightCommanderTM; Logic PD Inc.), whose intrinsic light engine had been replaced with our own multispectral LED light source containing four independently controlled LEDs (λ_{max} at 405nm, 455nm, 525nm and 630nm; Phlatlight PT-120 Series (Luminus Devices). Light from the LEDs was combined by a series of dichroic mirrors (ThorLabs), and directed onto the mirror device. The movie was presented using Python running PsychoPy Version 1.70.00 software. It featured mice moving around a behavioural arena including movement and looming of different sized objects (subtending visual angles ranging from 0.5° to 36°) at a range of orientations, speeds and contrasts (maximum Michelson contrast at 96%). The movie was presented at irradiance 0.81 W/m^2 with estimated retinal irradiance of $1x10^{13}$ rod equivalent photons/cm^2/s). The movie lacked differences in colour, and changes in irradiance across time were minimal (standard deviation of irradiance = 5.94%). Previous validations in *wild-type* mice have shown undetectable responses for presentations of de-focussed versions, indicating that most activity was elicited by changes in spatial patterns and object motion.

Behaviour
The modified light/dark box (dimensions: length=40cm width=40cm and height=30cm, open top) was made of Perspex and its walls painted white except for the two long sides of each arena, which were kept clear. Two identical infra-red lamps were placed centrally above each arena, to allow visualisation under dark conditions.
The visual stimuli were displayed from two 17-inch flat screen computer monitors (Acer V173b or Dell E173FP and ViewSonic matched for power by adjusting screen brightness) facing clear walls of each arena, using a DualHead2Go Digital Edition external multi-display adapter (Matrox Graphics Inc.). A variety of visual stimuli were generated using a custom written program and displayed on one monitor at a time. Stimuli included switching from 'black' (minimum brightness) to 'white', from steady grey to full screen flicker (Figure S4B to S4E) or square-wave gratings without an associated change in irradiance. The stimuli were presented when the mouse was in the middle of one half of the arena and the spatial frequency of gratings are reported for this viewing distance. Spatial frequency would become lower (by up to 2x) if the mouse moved towards the monitor and higher if it moved away. In addition a natural movie (colour rendition of an owl swooping in slow motion Figure S4F) was presented. Light intensity from 400-700nm was measured using a spectroradiometer (Bentham Instruments Ltd., UK or Cambridge Research Systems Ltd., UK). For full screen modulations, corneal irradiance for a

mouse looking directly at the screen was measured (for ease of comparison with other studies) by using a cosine diffuser light placed in the appropriate location in the arena.
Before the experimental period, mice were handled and habituated to their novel environment over 5 days, at the same time each day, by leaving them in the experimental box with their cagemates for 30 min. Following the habituation period, behaviour experiments were conducted over several weeks at the same time each day. Each group of mice was allowed to undergo only one testing condition per day. On each test day, mice were brought into the testing room in their home cages, allowed to accommodate to the testing room conditions for 30 minutes and then each mouse was tested individually. Mice were placed into the open field box (randomly to east or west half) and allowed to move freely between two arenas. All test trials were recorded under infra-red conditions through a camcorder fitted with an infra-red filter (λ=665nm). The box was thoroughly cleaned with 70% ethanol after each test trial and allowed to air-dry before next mouse was placed into the box.
A recording trial began after 3 minutes of habituation. Each trial run consisted of 5 minutes of control stimulus, following which a test stimulus was presented on a screen facing an arena that contained a mouse at this time point. The recorded trials were stored for off-line analysis using a video tracking software device (EthoVision® XT 10.1 Noldus, Tracksys Ltd., UK). We analysed distance travelled by each mouse in the entire box and outputted results in 30 second bins. The mouse's ability to detect the visual stimuli was assessed as a change in distance travelled in the 30s either side of test stimulus appearance. As we had no strong *a priori* expectation that stimuli would increase vs. decrease activity, we used a two-tailed paired *t* test to detect changes in locomotion. To account for habituation to the novel stimulus, we tested for statistically significant response across the group of treated mice in a single presentation for most tests here. The exception was the drifting grating, in which we explored the robustness of responses over repeated presentations in *wild-type* and rd^1 (treated and control) mice. We found that responses were retained over multiple presentations, but that high repeat numbers did not necessarily maximise the likelihood of detecting small effect sizes, as mouse behaviour appeared to change as they became increasingly accustomed to the task. Thus, e.g. we found that baseline activity progressively increased in both rd^1-grm6-*RHO* and rd^1-grm6-GFP animals and the magnitude of increases in activity decreased beyond 10 repeats (data not shown).

Supplemental references

S1. Lyubarsky, A.L., Daniele, L.L., and Pugh, E.N., Jr. (2004). From candelas to photoisomerizations in the mouse eye by rhodopsin bleaching in situ and the light-rearing dependence of the major components of the mouse ERG. Vision Res *44*, 3235-3251.

S2. Govardovskii, V.I., Fyhrquist, N., Reuter, T., Kuzmin, D.G., and Donner, K. (2000). In search of the visual pigment template. Vis Neurosci *17*, 509-528.

S3. Jacobs, G.H., and Williams, G.A. (2007). Contributions of the mouse UV photopigment to the ERG and to vision. Doc Ophthalmol *115*, 137-144.
